# Bacterial and Eukaryotic Small-Subunit Amplicon Data Do Not Provide a Quantitative Picture of Microbial Communities, but They Are Reliable in the Context of Ecological Interpretations

**DOI:** 10.1128/mSphere.00052-20

**Published:** 2020-03-04

**Authors:** Kasia Piwosz, Tanja Shabarova, Jakob Pernthaler, Thomas Posch, Karel Šimek, Petr Porcal, Michaela M. Salcher

**Affiliations:** a Centre Algatech, Institute of Microbiology, Czech Academy of Sciences, Třeboň, Czech Republic; b Department of Fisheries Oceanography and Marine Ecology, National Marine Fisheries Research Institute, Gdynia, Poland; c Institute of Hydrobiology, Biology Centre, Czech Academy of Sciences, České Budějovice, Czech Republic; d Limnological Station, Institute of Plant and Microbial Biology, University of Zurich, Kilchberg, Switzerland; e Faculty of Science, University of South Bohemia, České Budějovice, Czech Republic; University of Wisconsin-Madison

**Keywords:** CARD-FISH, amplicon sequencing, bacterial communities, bacterial community structure, bacterial dynamics, eukaryotic communities, eukaryotic community structure, eukaryotic dynamics, microbial abundance, microbial communities, microbial community structure, microbial dynamics

## Abstract

High-throughput sequencing (HTS) of gene amplicons is a preferred method of assessing microbial community composition, because it rapidly provides information from a large number of samples at high taxonomic resolution and low costs. However, mock community studies show that HTS data poorly reflect the actual relative abundances of individual phylotypes, casting doubt on the reliability of subsequent statistical analysis and data interpretation. We investigated how accurately HTS data reflect the variability of bacterial and eukaryotic community composition and their relationship with environmental factors in natural samples. For this, we compared results of HTS from three independent aquatic time series (*n* = 883) with those from an established, quantitative microscopic method (catalyzed reporter deposition-fluorescence *in situ* hybridization [CARD-FISH]). Relative abundances obtained by CARD-FISH and HTS disagreed for most bacterial and eukaryotic phylotypes. Nevertheless, the two methods identified the same environmental drivers to shape bacterial and eukaryotic communities. Our results show that amplicon data do provide reliable information for their ecological interpretations. Yet, when studying specific phylogenetic groups, it is advisable to combine HTS with quantification using microscopy and/or the addition of internal standards.

**IMPORTANCE** High-throughput sequencing (HTS) of amplified fragments of rRNA genes provides unprecedented insight into the diversity of prokaryotic and eukaryotic microorganisms. Unfortunately, HTS data are prone to quantitative biases, which may lead to an erroneous picture of microbial community composition and thwart efforts to advance its understanding. These concerns motivated us to investigate how accurately HTS data characterize the variability of microbial communities, the relative abundances of specific phylotypes, and their relationships with environmental factors in comparison to an established microscopy-based method. We compared results obtained by HTS and catalyzed reporter deposition-fluorescence *in situ* hybridization (CARD-FISH) from three independent aquatic time series for both prokaryotic and eukaryotic microorganisms (almost 900 data points, the largest obtained with both methods so far). HTS and CARD-FISH data disagree with regard to relative abundances of bacterial and eukaryotic phylotypes but identify similar environmental drivers shaping bacterial and eukaryotic communities.

## Abstract (precis)

High-throughput sequencing (HTS) of amplified fragments of rRNA genes provides unprecedented insight into the diversity of prokaryotic and eukaryotic microorganisms. Unfortunately, HTS data are prone to quantitative biases, which may lead to an erroneous picture of microbial community composition and thwart efforts to advance its understanding. These concerns motivated us to investigate how accurately HTS data characterize the variability of microbial communities, the relative abundances of specific phylotypes, and their relationships with environmental factors in comparison to an established microscopy-based method. We compared results obtained by HTS and catalyzed reporter deposition-fluorescence *in situ* hybridization (CARD-FISH) from three independent aquatic time series for both prokaryotic and eukaryotic microorganisms (almost 900 data points, the largest obtained with both methods so far). HTS and CARD-FISH data disagree with regard to relative abundances of bacterial and eukaryotic phylotypes but identify similar environmental drivers shaping bacterial and eukaryotic communities.

## INTRODUCTION

High-throughput sequencing (HTS) of 16S and 18S rRNA gene amplicons has revolutionized microbiome research in environmental samples (1) because it allows for the unprecedented time- and cost-effective processing of large numbers of samples, providing data at high taxonomic resolution (2). Presently, HTS methods are a commonly used tool to study microbial communities in diverse environments (3–8). They provide data on the abundance of sequence reads affiliated with a particular phylotype relative to the total numbers of reads in the sample. In many studies, such data are treated as actual proportions of the studied microbes in the original habitat, and they are used to generate correlation-based hypotheses on the importance of environmental factors for specific microbial groups (9). Unfortunately, biases introduced during sample processing, such as DNA extraction (10), PCR amplification (11), and uneven coverage of primers across phylogenetic groups (12), result in low quantitative accuracy of amplicon data with respect to translating the relative abundance of a specific phylotype in a sequencing library to its contribution in the samples. This shortcoming has been repeatedly documented using mock communities (13–15) but is largely ignored in the absence of an alternative. It is further aggravated by the uneven distribution of rRNA operons in prokaryotes (1 to 15 copies of rRNA genes) (16) and even more pronounced in eukaryotes (1 to 315,000 copies of rRNA genes) (17, 18). While current pipelines for the analysis of amplicon data minimize errors arising from sequencing and chimera formation (19–22), none of the postsequencing bioinformatic analysis can mitigate the previously listed biases. Thus, statistical analysis based on the relative abundances of individual microbial lineages derived solely from HTS data may hinder, or even misguide, our understanding of microbial community dynamics and functioning.

Catalyzed reporter deposition-fluorescence *in situ* hybridization (CARD-FISH) provides estimates of relative abundance (percent contribution to total bacterial or eukaryotic numbers) of individual microbial lineages defined based on rRNA gene phylogeny (23, 24), and it is a verified quantitative tool in numerous studies on bacterial and eukaryotic communities (25–30). The accuracy of CARD-FISH may be compromised by imperfect probe coverage and specificity, uneven permeabilization across phylogenetic groups, differences in the presence of endogenous peroxidases between phylogenetic groups and environmental samples, poor detection of low abundance or inactive community members, and difficulties in counting aggregated cells (23, 24). Despite these limitations, relative abundances obtained with CARD-FISH corresponded well to the actual proportions of phylotypes in mock communities (31). The main advantage of CARD-FISH over the HTS methods is that the relative abundance of a particular lineage can be evaluated independent of the other taxa in the samples. Moreover, the CARD-FISH procedure can be separately optimized for each target group (probe), which is not possible for PCR with primers that target many different templates. Finally, CARD-FISH results can be readily combined with results from direct enumeration methods, such as microscopy or flow cytometry, to provide absolute abundance estimates of microbial lineages in the samples. Nevertheless, the considerably less labor-intensive HTS methods have largely replaced CARD-FISH for studies of microbial communities (32).

Regardless of their complexity, mock communities used for an assessment of accuracy of HTS methods are substantially simpler than natural communities. So far, comparative studies of HTS versus microscopic counts from environmental samples have focused on few phylotypes and/or were based on a low number of samples, yielding rather inconsistent results (33–39). Moreover, in the case of eukaryotes, HTS data have usually been compared with abundance data derived from morphological analyses, even though the correspondence between morphotypes and phylotypes is limited by sequence availability in repositories (36, 40).

Despite these limitations, changes in and differences between microbial communities are typically deduced only from proportions of read numbers and so are the proposed external factors potentially affecting them. The latter are often elucidated from statistical multidimensional correlation models based on dissimilarity between the samples (41). Although significant correlations between HTS and CARD-FISH data (33) indicate that the similarity matrixes calculated from both types of data might also agree, to our knowledge this has not been tested on larger data sets so far.

We analyzed three data sets from distinct aquatic habitats that were investigated in parallel by using 454 pyrosequencing with general bacterial or eukaryotic primers (HTS data) and by CARD-FISH, to evaluate the correspondence of these methods in estimating the composition and variability of microbial communities and their relationships with external factors. The eukaryotic data set consisted of 31 samples collected weekly from the Baltic Sea, analyzed by HTS and by CARD-FISH using 11 probes. The bacterial data sets originate from two high-frequency sampling campaigns in contrasting freshwater environments. The first one included 24 samples collected from the humic Jiřická Pond (Czech Republic), analyzed by HTS (V1-V3 region) and by CARD-FISH using 20 probes. The second data set consisted of 24 samples from the oligo-mesotrophic Lake Zurich (Switzerland), analyzed by HTS (V3-V5 region) and by CARD-FISH using 5 probes. All together, this yielded 883 data points (278 for eukaryotes and 605 for bacteria), representing the largest comparative data set from environmental studies available so far.

## RESULTS AND DISCUSSION

### Direct comparison between HTS and CARD-FISH data (relative abundance/biovolume).

**FIG 1:**
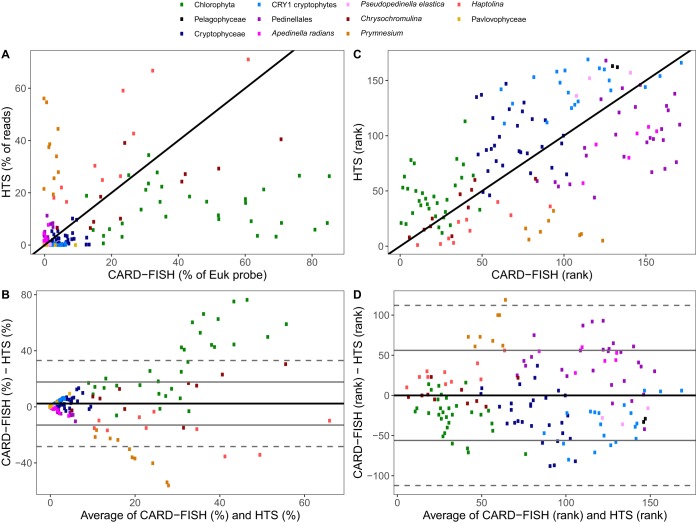
(A) Scatterplot of relative abundances of studied eukaryotic groups by 454 sequencing libraries (HTS) and CARD-FISH. (B) Scatterplot of differences between relative abundances of studied eukaryotic groups estimated by CARD-FISH and HTS against the average of the two values. (C) Scatterplot of ranked relative abundances of studied eukaryotic groups by HTS and CARD-FISH. (D) Scatterplot of differences between ranked relative abundances of studied eukaryotic groups estimated by CARD-FISH and HTS against the average of the two values. Black lines in panels A and C show a 1:1 relationship. Solid black lines in panels B and D show the average difference for the whole data set, solid gray lines show 1 standard deviation, and dashed gray lines show 2 standard deviations. Different eukaryotic groups are color coded. Individual plots for panels A and C are shown in Fig. S1 and S2 in the supplemental material, respectively.

The general agreement between the relative abundances of eukaryotic taxa determined by either CARD-FISH or HTS was poor (Fig. 1A; see Fig. S1 in the supplemental material). Correlations were not significant, and regression slopes differed significantly from the value of 1 for most of the lineages except for the haptophyte genus *Haptolina* (Table 1). HTS data of freshwater pelagic ciliates correlated better with biomass than with abundance (40), likely due to a higher number of rRNA genes in larger species (18). Unfortunately, of the studied nanoplanktonic groups, this was the case only for cryptophytes (Table 1; Fig. S2 and S3). The agreement between the two approaches was also analyzed by plotting differences between the relative abundances of CARD-FISH and HTS data points against their means (42). The average difference between these two values was greater than zero, both for abundance (Fig. 1B) and biomass (Fig. S2), providing further evidence for rather poor correspondence between HTS and CARD-FISH data (relative abundance or biomass) for specific eukaryotic phylotypes.

**TABLE 1:** Statistics for regressions and Spearman correlations between relative contributions to HTS or CARD-FISH data^*a*^

Group	Regression				Spearman correlation		*n*
	Adjusted *r*^2^	Slope	*P* (*r*)	*P* value for slope of 1	Rho	*P* (*S*)	
Eukaryotes—abundance							
Chlorophyta	−0.01	0.13	0.4420	<0.0001	0.10	0.5881	31
Pedinellales	0.04	0.31	0.1608	0.0029	0.36	0.0580	28
Cryptophyceae	−0.02	−0.32	0.5409	0.0167	0.02	0.8991	30
CRY1 cryptophytes	0.04	0.46	0.1869	0.1295	0.47	0.0320	21
*Chrysochromulina*	0.62	0.62	0.0042	0.0411	0.92	0.0005	10
*Haptolina*	0.70	0.69	0.0015	0.0661	0.92	0.0005	10
*Prymnesium*	−0.04	−0.13	0.7570	<0.0001	−0.42	0.2696	9
							
Eukaryotes—biomass							
Chlorophyta	−0.02	0.12	0.4980	<0.0001	0.16	0.3954	31
Pedinellales	0.11	0.42	0.0468	0.0084	0.45	0.0181	28
Cryptophyceae	0.23	0.95	0.0043	0.8612	0.54	0.0024	30
CRY1 cryptophytes	0.08	0.46	0.1138	0.0644	0.49	0.0258	21
*Chrysochromulina*	0.53	0.58	0.0101	0.0419	0.81	0.0082	10
*Haptolina*	0.53	0.62	0.0106	0.0774	0.75	0.0184	10
*Prymnesium*	−0.14	−0.01	0.9410	0.0005	−0.23	0.5517	9
							
Bacteria—Jiřická Pond							
*Alphaproteobacteria*	−0.01	−0.38	0.4043	0.0053	−0.27	0.2094	24
*Actinobacteria*	0.48	0.47	<0.0001	<0.0001	0.72	0.0001	24
“*Ca*. Nanopelagicales”	0.90	0.74	<0.0001	<0.0001	0.93	<0.0001	24
Luna-2 cluster, *Actinobacteria*	0.17	0.51	0.0279	0.0354	0.39	0.0681	23
“*Ca*. Nanopelagicus”	0.71	0.73	<0.0001	0.0146	0.84	<0.0001	23
“*Ca*. Planktophila versatilis”	0.20	0.83	0.0374	0.6432	0.81	<0.0001	18
*Bacteroidetes*	0.23	0.52	0.0100	0.0149	0.45	0.0277	24
*Betaproteobacteria*	0.35	0.59	0.0015	0.0187	0.56	0.0055	24
Uncult. lineage GKS98 *Betaproteobacteria*	0.46	0.71	0.0002	0.0758	0.71	0.0002	24
*Limnohabitans* cluster LimA	0.59	0.73	<0.0001	0.0408	0.76	<0.0001	24
*Limnohabitans* cluster LimB	−0.05	−0.08	0.8340	0.0078	0.59	0.0036	22
*Limnohabitans* clusters LimBCD	0.03	−0.19	0.2100	<0.0001	−0.39	0.0808	21
All *Limnohabitans*	0.35	0.66	0.0013	0.0684	0.60	0.0020	24
*Methylophilaceae*	0.19	0.64	0.0190	0.1770	0.31	0.1433	24
*Polynucleobacter* clusters PnecABD	0.70	1.04	<0.0001	0.7979	0.89	<0.0001	24
*Polynucleobacter* cluster PnecC	0.76	0.72	<0.0001	0.0034	0.57	0.0046	24
“*Ca.* Methylopumilus turicensis*”*	−0.03	0.15	0.5310	0.0020	−0.15	0.4739	24
*Opitutae*	0.65	0.98	<0.0001	0.8775	0.81	<0.0001	24
*Verrucomicrobia* (excluding *Opitutae*)	0.04	0.69	0.1795	0.5489	0.01	0.9597	24
All *Verrucomicrobia*	0.67	1.28	<0.0001	0.1508	0.70	0.0002	24
							
Bacteria—Lake Zurich							
*Bacteroidetes*	0.67	0.82	<0.0001	0.1430	0.75	<0.0001	24
“*Ca*. Nanopelagicales”	−0.02	−0.06	0.44	<0.0001	−0.17	0.4241	24
“*Ca*. Methylopumilus planktonicus”	−0.03	0.12	0.56	0.0002	0.02	0.9164	24
*Polynucleobacter* cluster PnecB	0.17	0.73	0.025	0.3874	0.52	0.0100	24

a Regression statistics include adjusted *r*^2^, slope value, and significance level (*P* [*r*]) and Spearman correlation statistics include rho and significance level (*P* [*S*]) between relative contributions (percentages) to HTS or CARD-FISH data. A *P* value slope of 1 indicates a significance level against the desired value of 1, while a *P* value slope of >0.05 indicates that the slope is not significantly different from 1. *n*, number of data points for each group. Uncult., uncultured.

10.1128/mSphere.00052-20.1 FIG S1 Individual scatter plots of relative abundances of studied eukaryotic groups by 454 sequencing libraries (HTS) and CARD-FISH. Black lines show a 1:1 relationship. Axis scales differ between the panels. Download FIG S1, PDF file, 0.1 MB. Copyright © 2020 Piwosz et al. 2020 Piwosz et al. This content is distributed under the terms of the Creative Commons Attribution 4.0 International license.

10.1128/mSphere.00052-20.2 FIG S2 (A) Scatterplot of relative biovolumes of studied eukaryotic groups from 454 sequencing libraries (HTS) and CARD-FISH. (B) Scatterplot of ranked relative biovolumes of studied eukaryotic groups from HTS and CARD-FISH. (C) Scatterplot of differences between relative biovolumes of studied eukaryotic groups estimated by CARD-FISH and HTS against the average of the two values. (D) Scatterplot of differences between ranked relative biovolumes of studied eukaryotic groups estimated by CARD-FISH and HTS against the average of the two values. Black lines in panels A and B show a 1:1 relationship. Solid black lines in panels C and D show average differences for the whole data set, solid gray lines show 1 standard deviation, and dashed gray lines show 2 standard deviations. Different eukaryotic groups are color coded. Individual plots for panels A and B are shown in Fig. S3 and S4, respectively. Download FIG S2, PDF file, 0.2 MB. Copyright © 2020 Piwosz et al. 2020 Piwosz et al. This content is distributed under the terms of the Creative Commons Attribution 4.0 International license.

10.1128/mSphere.00052-20.3 FIG S3 Individual scatterplots of relative biovolumes of studied eukaryotic groups from 454 sequencing libraries (HTS) and CARD-FISH. Black lines show a 1:1 relationship. Axis scales differ between the panels. Download FIG S3, PDF file, 0.1 MB. Copyright © 2020 Piwosz et al. 2020 Piwosz et al. This content is distributed under the terms of the Creative Commons Attribution 4.0 International license.

The agreement between HTS and CARD-FISH was better when samples in each data set were ranked from highest to lowest relative abundance (Fig. 1C and D; Fig. S4 and S5). The Spearman rank correlations were strong for most eukaryotic groups and significant for biovolume (Table 1). These findings speak for the use of nonparametric methods for the analysis of HTS data (9).

10.1128/mSphere.00052-20.4 FIG S4 Individual scatterplots of rank relative biovolumes of studied eukaryotic groups from 454 sequencing libraries (HTS) and CARD-FISH. Samples with zeroes in both CARD-FISH and HTS data were removed, because they were arbitrarily ranked. Black lines show a 1:1 relationship. Axis scales differ between the panels. Download FIG S4, PDF file, 0.1 MB. Copyright © 2020 Piwosz et al. 2020 Piwosz et al. This content is distributed under the terms of the Creative Commons Attribution 4.0 International license.

10.1128/mSphere.00052-20.5 FIG S5 Individual scatterplots of rank relative abundances of studied eukaryotic groups from 454 sequencing libraries (HTS) and CARD-FISH. Samples with zeroes in both CARD-FISH and HTS data were removed, because they were arbitrarily ranked. Black lines show a 1:1 relationship. Axis scales differ between the panels. Download FIG S5, PDF file, 0.1 MB. Copyright © 2020 Piwosz et al. 2020 Piwosz et al. This content is distributed under the terms of the Creative Commons Attribution 4.0 International license.

**FIG 2:**
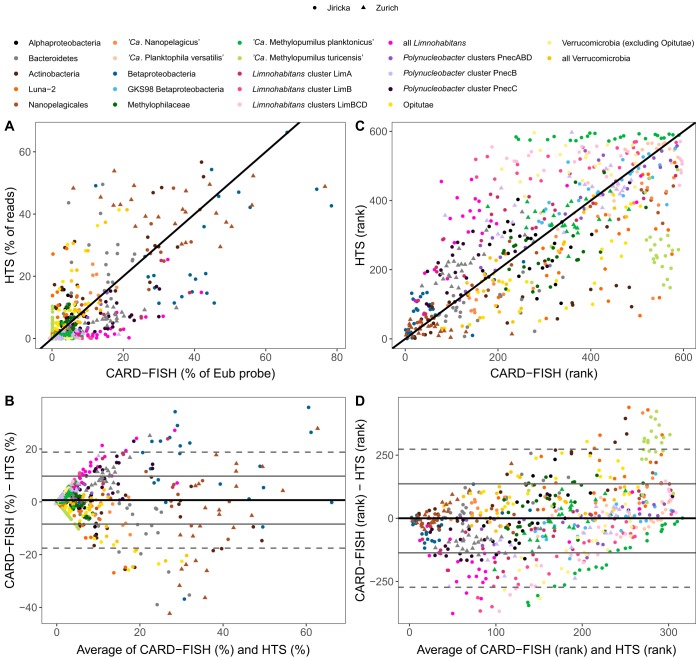
(A) Scatterplot of relative abundances of studied bacterial groups (pooled data sets from both lakes) by 454 sequencing libraries (HTS) and CARD-FISH. (B) Scatterplot of differences between relative abundances of studied bacterial groups estimated by CARD-FISH and HTS against the average of the two values. (C) Scatterplot of ranked relative abundances of studied bacterial groups by HTS and CARD-FISH. (D) Scatterplot of differences between ranked relative abundances of studied bacterial groups estimated by CARD-FISH and HTS against the average of the two values. Black lines in panels A and C show a 1:1 relationship. Solid black lines in panels B and D show average differences for the whole data set, solid gray lines show 1 standard deviation, and dashed gray lines show 2 standard deviations. Different bacterial groups are color coded, and lakes of sample collection are indicated by shape. Individual plots for panels A and C are shown in Fig. S6 and S7, respectively.

In general, the agreement between the two approaches was better for bacteria than for eukaryotes, for both relative and rank data (Fig. 2; Table 1). Most bacterial groups were underrepresented in HTS data compared to CARD-FISH data, e.g., different lineages of *Limnohabitans*, “*Candidatus* Methylopumilus planktonicus,” *Polynucleobacter* subclusters B and C, “*Ca*. Planktophila vernalis”, the uncultivated *Betaproteobacteria* lineage GKS98 and *Verrucomicrobia* (without *Opitutae*). In contrast, “*Ca*. Methylopumilus turicensis.” *Opitutae*, or the Luna-2 cluster of *Actinobacteria* were overrepresented in HTS data compared to CARD-FISH data (Table 1; Fig. S6). Such phylotype-dependent agreement between the HTS and CARD-FISH data was also observed in marine mesocosms (43), indicating that a simple interpretation of HTS data in terms of relative abundance should be avoided for bacteria as well.

10.1128/mSphere.00052-20.6 FIG S6 Individual scatterplots of relative abundances of studied bacterial groups by 454 sequencing libraries (HTS) and CARD-FISH. Black lines show a 1:1 relationship. Axis scales differ between the panels. Download FIG S6, PDF file, 0.3 MB. Copyright © 2020 Piwosz et al. 2020 Piwosz et al. This content is distributed under the terms of the Creative Commons Attribution 4.0 International license.

10.1128/mSphere.00052-20.7 FIG S7 Individual scatterplots of rank relative abundances of studied bacterial groups from 454 sequencing libraries (HTS) and CARD-FISH. Black lines show a 1:1 relationship. Axis scales differ between the panels. Download FIG S7, PDF file, 0.3 MB. Copyright © 2020 Piwosz et al. 2020 Piwosz et al. This content is distributed under the terms of the Creative Commons Attribution 4.0 International license.

**FIG 3:**
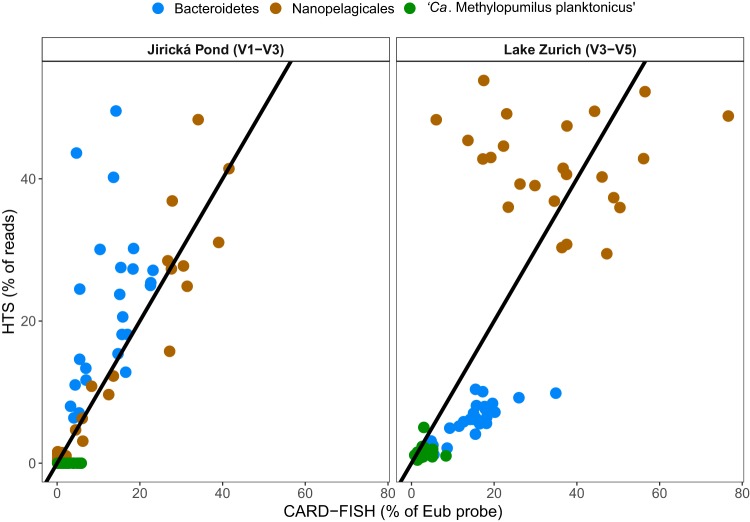
Scatterplots of relative abundances of the same bacterial groups by 454 sequencing libraries (HTS) and CARD-FISH in Jiřická Pond and Lake Zurich. HTS data for each lake were generated with a different primer set (Table S1). Black lines show 1:1 relationship.

Considerably higher agreement between the two approaches was obtained when rank-transformed relative abundances were used (Fig. 2C and D), especially for “*Ca*. Nanopelagicus,” *Polynucleobacter* cluster A, “*Ca*. Planktophila vernalis,” and the GKS98 lineage (Table 1; Fig. S7). Interestingly, there was a site-specific difference for “*Ca.* Nanopelagicales” (*Actinobacteria*), which yielded a good correspondence for samples from Jiřická Pond but a poor one for Lake Zurich (Fig. 3). The opposite was observed for “*Ca*. Methylopumilus planktonicus,” which was not detected at all by HTS in Jiřická Pond. These incongruities could not be explained by discrepancies in the respective coverage of the different primer sets used for generating the two data sets, as both primer pairs displayed very good *in silico* coverages of these two bacterial groups (90.9% versus 92.9% for “*Ca*. Nanopelagicales” and 98.3% versus 90.9% for “*Ca*. Methylopumilus planktonicus”) (Tables S1 and S2).

10.1128/mSphere.00052-20.9 TABLE S1 List of primers used in this study. Download Table S1, PDF file, 0.3 MB. Copyright © 2020 Piwosz et al. 2020 Piwosz et al. This content is distributed under the terms of the Creative Commons Attribution 4.0 International license.

The overall poor agreement between relative abundances derived from HTS and CARD-FISH could not be explained by different specificities of probes and primers. On the one hand, the 10-fold-higher HTS-derived relative abundance of the haptophyte genus *Prymnesium* (Fig. S1), detection of which is a major concern in areas where it forms toxic blooms (44), could be attributed to the lower coverage of the group by the CARD-FISH probe than by the primer (70.3% and 87.2%, respectively) (Tables S1 and S2). On the other hand, Pavlovophyceae, whose coverages by probe and primers are very similar (87.2% and 83.0%, respectively) (Tables S1 and S2), were completely undetected by sequencing (Fig. S1). All bacterial lineages that were overrepresented in the HTS data set displayed comparable or even slightly higher *in silico* coverages of CARD-FISH probes than of HTS primers (“*Ca*. M. turicensis,” *Opitutae*, “*Ca*. Nanopelagicus,” and *Actinobacteria* of the Luna-2 lineage) (Tables S1 and S2). Likewise, some lineages that were overrepresented by CARD-FISH had a much higher coverage with general PCR primers (e.g., *Verrucomicrobia* excluding *Opitutae*, the *Betaproteobacteria* lineage GKS98), while coverage was very similar in others (e.g., “*Ca*. Planktophila vernalis,” *Polynucleobacter* lineage PnecC) (Tables S1 and S2). In fact, agreement between the differences in coverage by HTS primers (80%) and CARD-FISH probes (100%) and of the relative abundances detected by either approach was found only in *Polynucleobacter* lineage PnecB. In any case, differences in coverage between probes and primers cannot explain phenomena such as the good agreement between HTS and CARD-FISH for “*Ca.* Nanopelagicales” in Jiřická Pond but not in Lake Zurich (Fig. 3), as the group coverages of both HTS primer sets were very similar (92.2% and 90.9%, respectively) (Table S1) and much higher than that of the probe (68.0%) (Table S2). This indicates the importance of other, unknown biases besides primer and probe coverage.

10.1128/mSphere.00052-20.10 TABLE S2 List of probes, helpers, and competitors used in this study. HB (%), concentration of formamide in the hybridization buffer; T, hybridization temperature (washing temperature was 2°C higher); Probe, coverage (in percent of targeted sequences) of the probe; Primers 1, coverage of primers TAReuk454FWD1 and TAReukREV3 (eukaryotes) or 341F and 907R (bacteria, Lake Zurich); Primers 2, coverage of primers TAReuk454FWD1 and HaptoR1 (eukaryotes) or 27F andUni522R (bacteria, Jiřická Pond). The numbers are based on search in the reference Silva database release 132, conducted on 23 November 2019. Download Table S2, PDF file, 0.6 MB. Copyright © 2020 Piwosz et al. 2020 Piwosz et al. This content is distributed under the terms of the Creative Commons Attribution 4.0 International license.

Limitations connected with PCR biases and imperfect probe and primer coverage and specificity may be potentially overcome by the use of taxonomically profiled metagenomic data to estimate relative abundances of specific groups, but so far tests with mock communities have suggested otherwise (45). Recently, an addition of known amounts of Escherichia coli cells has been proposed as an internal standard for amplicon read normalization in freshwater bacterial communities (31). This approach provided substantially improved estimates for relative changes of phylotype contributions between samples compared to those of nonnormalized reads. It would be valuable to explore its applicability for eukaryotes as well.

### Comparison of statistical models.

The above results provide further evidence that the relative abundances of phylotypes as derived from HTS data should not be directly translated into the proportions or biovolumes of cells from these lineages in a sample. However, the generally better agreement of rank data suggests that nonparametric distance-based ordination methods might be appropriate for the analysis of HTS data, e.g., to study differences between microbial communities in different habitats (9). To test this hypothesis, we calculated Bray-Curtis distance matrices for both CARD-FISH and HTS data for all data sets and compared those using two-tailed Mantel tests. For eukaryotes, we found a weak but significant nonparametric correlation between relative abundances derived from HTS and both relative abundances (Spearman’s rho = 0.1533, *P* < 0.0001) and relative biovolumes determined by CARD-FISH (rho = 0.1083, *P* = 0.0211). Similar results were obtained for bacteria from Lake Zurich, where relative abundances of HTS and CARD-FISH also correlated weakly but significantly (rho = 0.2025, *P* = 0.0005). In contrast, very strong and significant correlations of HTS and CARD-FISH data were observed for bacteria from Jiřická Pond (rho = 0.8104, *P* < 0.0001).

Multivariate methods are often used to generate correlation-based hypotheses about the respective importance of different environmental variables for microbial community dynamics. We analyzed both CARD-FISH and HTS data with distance-based linear models (DistML). The agreement was very good for the eukaryotic data set, as in both cases DistML pointed to soluble reactive phosphorus (SRP) as the only explanatory variable (Table 2). However, this result was largely driven by a single outlier sample, for which we observed elevated SRP concentrations and a massive bloom of the dinoflagellate Heterocapsa triquetra (46). The abundance of nanophytoplankton was substantially lower in this sample, and the sequencing library was dominated by reads from the dinoflagellate. When this sample was excluded from the analysis, a combination of SRP and temperature best explained the variability of CARD-FISH relative abundance data, but there was no significant model for the HTS data. Interestingly, this agreed with the CARD-FISH biovolume data, for which a significant model was not found in both cases (Table 2). This imperfect agreement between the models can be partially attributed to the fact that eukaryotic abundance and biovolume may respond differently to changing conditions (46). In general, it seems that statistical models calculated from HTS and CARD-FISH data may identify the same environmental drivers affecting eukaryotic microbial communities, but it is advisable to combine and calibrate HTS with microscopic methods during experiments aimed to test hypotheses derived from statistical models on observational data.

**TABLE 2:** DistML models for the eukaryotic data set calculated from HTS and CARD-FISH data (relative abundance and biovolume)

Sample(s)	Variable^*a*^	DistML model for data calculated from:		
		HTS: relative abundance [*P* value (% explained variation)]	CARD-FISH	
			Relative abundance [*P* value (% explained variation)]	Relative biovolume
All samples	SRP	0.0276 (13.5)	0.0165 (12.8)	No significant model
No outlier sample	SRP	No significant model	0.0015 (14.2)	No significant model
	Temp		0.0072 (14.5)	

a SRP, soluble reactive phosphorus.

Excellent agreement between HTS and CARD-FISH was found in the bacterial data sets. In the case of Lake Zurich, the patterns of relative abundance derived by either approach pointed to temperature and abundance of virus-like particles (VLP) as the best explanatory variables (Table 3). For Jiřická Pond, all models included dissolved organic carbon, total phosphorus, water residence time, and either dissolved nitrogen (HTS) or chlorophyll *a* (CARD-FISH) (Table 3). This almost perfect correspondence indicates that use of distance-based multivariate analyses for bacterial amplicon HTS data allows for the generation of models and hypotheses similar to those obtained from relative abundance data from CARD-FISH.

**TABLE 3:** DistML models for bacterial data sets calculated from HTS and CARD-FISH data

Sampling site	Variable^*a*^	DistML model for data calculated from:	
		HTS: relative abundance [*P* value (% explained variation)]	CARD-FISH: relative abundance [*P* value (% explained variation)]
Lake Zurich	Temp	0.0001 (42.9)	0.0097 (18.9)
	VLP	0.0092 (14.1)	0.05 (11.3)
			
Jiřická Pond	WRT 0.5m	0.0001 (29.4)	0.0004 (10.8)
	DOC	0.0001 (18.9)	0.0001 (37.3)
	TP	0.0002 (14.9)	0.0009 (15.9)
	DN	0.0061 (6.4)	
	Chl-a 0.5m		0.0231 (5.1)

a VLP, abundance of virus-like particles; WRT 0.5m, water residence time at 0.5-m depth; DOC, dissolved organic carbon; TP, total phosphorus; DN, dissolved nitrogen; Chl-a 0.5m, chlorophyll *a* at 0.5-m depth.

### Caveats of the study.

Our HTS data are based on the pyrosequencing 454 method (Roche) that has been replaced by newer platforms that provide sequencing depth orders of magnitude higher, such as Illumina or Oxford Nanopore. However, as the main biases arise from DNA extraction, PCR amplification, and uneven 16S and 18S rRNA gene copy numbers (47), these newer methods will not necessarily improve the quantitative accuracy of the sequencing data, as shown with mock communities sequenced using Illumina (13–15) and Oxford Nanopore (48, 49) platforms. In contrast, the relative abundances of sequences obtained from the same samples by both pyrosequencing and Illumina correlated very strongly (*r*^2^ > 0.99) (50). It has been shown that 3,000 reads per sample are sufficient to capture >90% of alpha-diversity in samples from freshwater lakes and to reveal beta-diversity patterns (2). In our study, the lowest number of reads per sample was 1,724 for the eukaryotic data set (average, 4,742), 34,020 for the bacterial data set from Jiřická Pond (average, 68,450), and 3,877 for the bacterial data set from Lake Zurich (average, 11,863). Finally, although it cannot be completely excluded that we missed some reads of phylotypes targeted by the CARD-FISH probes by using 454 pyrosequencing instead of Illumina, rarefaction analysis indicated that most would belong to the rare biosphere (Fig. S8). All of these suggest that sequencing depth was sufficient to capture most of the diversity in our samples. Thus, our conclusions are not considerably affected by lower sequencing depth and likely apply to all nonnormalized PCR-based sequencing methods.

10.1128/mSphere.00052-20.8 FIG S8 Individual rarefaction curves for samples from the Baltic Sea (eukaryotes) (A), Jiřická Pond (B), and Lake Zurich (C). Download FIG S8, PDF file, 0.2 MB. Copyright © 2020 Piwosz et al. 2020 Piwosz et al. This content is distributed under the terms of the Creative Commons Attribution 4.0 International license.

Mock community studies have pointed out the importance of PCR conditions for the accurate recovery of bacterial lineages (primer choice, annealing conditions, polymerase type, and number of cycles) (13–15, 47). The PCR conditions used here were standard at the time of the study (51) but have since then been shown to decrease quantitative accuracy and increase chimera formation (52). However, only very few chimeras were detected in our data sets (eukaryotes, 1.2% of operational taxonomic units [OTUs]; bacteria in Jiřická Pond, 9.8% of OTUs; bacteria in Lake Zurich, 5.7% of OTUs). The phylotype-dependent agreement between HTS and CARD-FISH data (Fig. 1 and 3; Fig. S1 to S7) indicates that a template-dependent PCR bias might have dominated in our samples (47). Finally, it has been shown that even optimized PCR conditions do not reproduce original communities with perfect qualitative accuracy (13, 47). All together, although the use of fewer PCR cycles and proofreading polymerase might have arguably improved the agreement (correlations) between the two methods, our main conclusions likely remain unaffected.

### Conclusions.

Our study presents the largest data set comparing HTS and CARD-FISH data from natural samples (almost 900 data points) to date. It expands previous observations derived from mock communities, i.e., that the relative abundances of specific phylotypes obtained by HTS may not necessarily correspond to their relative abundances in the original samples. Despite this limitation, we show that nonparametric distance-based multivariate analyses based on HTS and CARD-FISH data often agree and thus seem to allow for reliable ecological interpretations of the relationship between microbial community structure and environmental parameters. This appears to work especially well under conditions that cause substantial changes in community composition, as observed for Jiřická Pond. In summary, it appears that studies focusing on the relationship of whole microbial communities with environmental variables can perhaps rely solely on HTS data. In contrast, we recommend that sequence-based community analysis (optimally using internal standards) be combined with CARD-FISH when aiming at more accurate estimates of abundances or biomass of specific bacterial taxa or when studying eukaryotes.

## MATERIALS AND METHODS

### Eukaryotes. (i) Sample collection.

Coastal waters of the Gulf of Gdańsk (Baltic Sea) were sampled weekly from 12 April to 7 November 2012. Twenty liters of surface seawater was prefiltered through a 20-μm net and transported to the laboratory within 15 min in a darkened, closed container. Temperature and salinity were measured *in situ* with an InoLab probe (WTW).

Biomass for amplicon sequencing was collected from 0.8 to 4.6 liters of sampled water filtered onto polyethersulfone filters (0.22-μm pore size, 47-mm diameter; GPWP04700; Millipore-Merck KGaA, Darmstadt, Germany) under aseptic conditions. The filters were stored at –80°C.

For CARD-FISH, a 200-ml subsample was fixed by the Lugol-formalin-sodium thiosulfate method recommended for preservation of fragile protists (53). Fixed samples were stored in the dark at 4°C for 16 h, filtered onto white polycarbonate filters (0.8-μm pore size, 47-mm diameter; Cyclopore; Whatmann, Maidstone, UK) under low pressure (<2 × 10^4^ Pa), rinsed with 50 ml of sterile MilliQ water, air dried, and stored at –20°C.

### (ii) Environmental variables.

Concentrations of dissolved inorganic nitrogen (DIN) as well as soluble reactive phosphorus (SRP) and dissolved silicate (DSi) were determined by methods recommended for the Baltic Sea (54). For this purpose, 500 ml of water was collected in an acid-cleaned container, frozen at –20°C, and analyzed within 1 month.

### (iii) DNA extraction and sequencing.

DNA was extracted using a PowerWater DNA isolation kit (MO BIO Laboratories, Inc., Carlsbad, CA, USA). Extracted DNA samples were processed by Research and Testing Laboratories (Lubbock, TX, USA). The V-4 fragments of 18S rRNA genes were amplified with TAReuk454FWD1 and TAReukREV3 (see Table S1 in the supplemental material). Amplifications were performed in 25-μl reaction volumes with recombinant Hot Start *Taq* polymerase (Qiagen HotStarTaq master mix; Qiagen, Inc., Valencia, CA, USA), 1 μl of each 5 μM primer, and 1 μl of template on ABI Veriti thermocyclers (Applied Biosytems, Carlsbad, CA, USA) under the following thermal profile: 95°C for 5 min, followed by 10 cycles of 94°C for 30 s, 57°C for 45 s, and 72°C for 1 min and then 25 additional cycles of 94°C for 30 s, 45°C for 45 s, and 72°C for 1 min, and a final 2-min extension at 72°C (51). As the reverse primer TAReukREV3 poorly targets haptophytes, we additionally sequenced samples with high haptophyte abundance (23 May to 30 July) using the reverse primer HaptoR1 (Table S1) under the following thermal profile: 95°C for 5 min, followed by 35 cycles of 94°C for 30 s, 55°C for 45 s, and 72°C for 1 min, and a final 2-min extension at 72°C (55). The amplicons were sequenced using the Roche 454 GS FLX Titanium platform with an average sequencing depth of 10,000 raw reads per sample.

### (iv) CARD-FISH.

The CARD-FISH procedure was performed with Alexa 488-labeled tyramides (Molecular Probes, Thermo Fisher Scientific, Waltham, MA, USA), as previously described (56), and analyzed manually using 10 to 20 microphotographs randomly taken by epifluorescence microscopy at ×1,000 magnification (AxioVision.M1; Carl Zeiss, Jena, Germany). Biovolume was estimated by multiplying cell abundance by average cell volume, which was calculated based on manual measurements of cell width and length and assuming the cell shape to be prolate spheroid, as described by Piwosz in 2019 (46). The relative abundance of an individual lineage was calculated as the proportion of cells hybridized with the specific probe to that of cells hybridized with the general eukaryotic probe. A full list of applied probes (*n* = 11) can be found in Table S2.

### (v) Bioinformatics analysis.

Sequences were analyzed using a custom-made pipeline as previously described (57). Raw sff flowgrams were denoised using AmpliconNoise (52). The demultiplexed and primer-free reads were quality filtered and trimmed to a length of 250 bp using USEARCH (58) (bases with a Phred score of <30 were trimmed), and chimeric sequences were discarded with UCHIME (59). OTUs were clustered by average linkage at similarity levels of 97% upon the pairwise alignment by the Needleman-Wunsch algorithm. The most closely related sequence for each OTU was identified using pairwise alignment to the curated eukaryotic PR2 reference data (60), and the corresponding taxonomic information, together with the coverage and dissimilarity to the query sequence, was assigned. The final number of reads in samples ranged from 1,707 to 15,233.

### Bacteria. (i) Sample collection.

Jiřická Pond is a shallow, humic pond in the southern region of the Czech Republic and is characterized by short-term flooding events, severely shortening its hydraulic retention time, which triggers sudden fluctuations in microbial communities (61, 62). An intensive sampling campaign took place between 5 May and 27 June 2014, with samples taken three times per week. Water samples from a 0.5-m depth were taken with a Friedinger sampler and split into subsamples. Samples for prokaryotic cell counts and CARD-FISH were fixed with formalin (2%, vol/vol). Fixed subsamples for CARD-FISH were filtered onto white polycarbonate filters within 16 h after sampling (0.2-μm pore size, 47-mm diameter; Millipore-Merck KGaA, Darmstadt, Germany) and stored at –20°C. Samples for enumeration of virus-like particles (VLP) were fixed with glutaraldehyde (0.5%, vol/vol) for 10 min, flash-frozen in liquid nitrogen, and stored at –80°C until evaluation via flow cytometry (63). Prokaryotic biomass for amplicon sequencing was collected on polysulfone Sterivex filters (0.22-μm pore size; Millipore-Merck KGaA, Darmstadt, Germany). Additionally, 2 liters of water was taken for chemical analyses. These samples were delivered in a ThermoBox to the laboratory and analyzed within 24 h.

For Lake Zurich, a longitudinal transect of eight sampling stations along Lake Zurich (26) and the connected Upper Lake was sampled in summer 2010 (27 and 28 July). Vertical profiles of temperature, conductivity, turbidity, and concentrations of oxygen and chlorophyll *a* (differentiating pigments of diatoms and Planktothrix rubescens [64]) were recorded with a YSI multiprobe (model 6600; Yellow Springs Instruments, Yellow Springs, OH, USA) and a bbe FluoroProbe (TS-16-12; bbe Moldaenke GmbH, Schwentinental, Germany), respectively. Water samples from three different depths representing the epilimnion (2 to 5 m), metalimnion (12.5 to 15 m), and hypolimnion (20 m) were taken with a Friedinger sampler for each sampling station and split in subsamples for (i) total counts of prokaryotes, (ii) VLP, (iii) CARD-FISH analyses, (iv) prokaryotic biomass for amplicon sequencing, and (v) chemical analyses. Subsamples (i) were fixed with formalin (2% vol/vol) and stored at 4°C. Subsamples (ii) and (iii) were processed as described above for Jiřická Pond. Subsamples (iv) (600 ml) were filtered on the same day onto polysulfone filters (0.2-μm pore size, 47-mm diameter; Millipore-Merck KGaA, Darmstadt, Germany) and stored at –80°C.

### (ii) Environmental variables.

For Jiřická Pond, water temperature, water retention time, and dissolved organic carbon (DOC) and chlorophyll *a* concentrations at 0.5-m depth were assessed as previously described (61). Concentrations of nitrate, nitrite, and ammonium ions were determined by ion chromatography (IC25; Dionex, USA). Values of total and dissolved phosphorus (TP and DP, respectively) were measured as described by Porcal and Kopáček (62). Dissolved nitrogen (DN) concentrations were obtained using a vario TOC cube (Elementar, Germany).

For Lake Zurich, concentrations of TP, DP, DOC, and different nitrogen species were determined by standard techniques by the Zurich Water Supply Company.

### (iii) DNA extraction and sequencing.

For Jiřická Pond, nucleic acid isolation was conducted using phenol-chloroform-isoamyl alcohol extraction according to a previously described protocol (65). The variable regions V1-V3 of the 16S rRNA gene were amplified with primers 27Fand Uni522R (Table S1). A single-step PCR using a HotStarTaq Plus master mix kit (Qiagen, Inc., Valencia, CA, USA) was conducted using the following profile: 94°C for 3 min, followed by 28 cycles of 94°C for 30 s, 53°C for 40 s, and 72°C for 1 min, and a final elongation step at 72°C for 5 min. After PCR, all amplicon products were mixed in equal concentrations and purified using Agencourt Ampure beads (Agencourt Bioscience Corporation, MA, USA). The amplicons were sequenced using the Roche 454 GS FLX Titanium platform at MR DNA laboratory (Shallowater, TX, USA) with an overage sequencing depth of 50,000 raw reads per sample.

For Lake Zurich, DNA was isolated with a PowerWater DNA isolation kit (MO BIO Laboratories, Inc., Carlsbad, CA, USA). Extracted DNA samples were processed by Research and Testing Laboratories (Lubbock, TX, USA). V3-V5 fragments of 16S rRNA genes were amplified with primers 341F and 907R (Table S1). Amplifications were performed in 25-μl reaction mixtures with recombinant HotStart *Taq* polymerase (Qiagen HotStarTaq master mix; Qiagen, Inc., Valencia, CA), 1 μl of each 5 μM primer, and 1 μl of template on ABI Veriti thermocyclers (Applied Biosystems, Carlsbad, CA) under the following thermal profile: 95°C for 5 min, followed by 35 cycles of 94°C for 30 s, 54°C for 40 s, and 72°C for 1 min, followed by a final 10-min extension at 72^°^C. The amplicons were sequenced using the Roche 454 GS FLX Titanium platform with an average sequencing depth of 10,000 reads per sample.

### (iv) CARD-FISH.

CARD-FISH for bacteria was carried out as previously described with fluorescein-labeled tyramides (66) and analyzed with a fully automated microscope (AxioImager.Z1; Carl Zeiss) as outlined by Salcher et al. (67). The relative abundance of an individual lineage was calculated as the proportion of cells hybridized with the specific probe to that of cells hybridized with the general bacterial probe. A full list of applied probes (*n* = 20) is provided in Table S2.

### (v) Bioinformatics analysis.

The demultiplexed and primer-free reads were quality filtered and trimmed to a length of 350 bp according to quality report using USEARCH (58). Chimeric sequences were detected and discarded using UCHIIME (59). OTUs were clustered at similarity levels of 97% using the UPARSE-OTU algorithm (68). A taxonomical assignment for representative sequences for each OTU was done with a parallel BLAST search against the SILVA-database SSURef_NR99_132 (69). The final numbers of reads in samples ranged from 34,020 to 104,696 for samples from Jiřická Pond and from 3,877 to 25,031 for samples from Lake Zurich. Data sets were rarefied to the smallest sample prior to statistical analysis.

### Statistical analysis.

The read numbers of all OTUs affiliated with lineages that corresponded to those targeted by probes were pooled, and their percent contributions to the total number of reads in each sample were compared with relative abundances (and biovolumes for eukaryotes) estimated by CARD-FISH. Relative abundances and biovolumes of individual eukaryotic or bacterial lineages were calculated as percentages of all hybridized cells (i.e., counts with general eukaryotic [Euk516] or bacterial [EubI-III] probes, respectively). The agreement between the two methods was assessed using graphical techniques, as described by Bland and Altman (42). The same methods were used to compare sample rankings by HTS and CARD-FISH. In addition, linear regressions and Spearman correlations were calculated between relative abundances derived from HTS and CARD-FISH. Multiple null values in data obtained using one of the approaches were pooled, and an average value was calculated for the second approach (i.e., if in HTS data there were three data points with null values, an average value for CARD-FISH data for these three points was calculated). The calculations were performed in the R environment version 3.3.3 (70), and the figures were prepared using functions from the ggplot2 package version 3.2.0 (71) and the ggpubr package version 0.2.1. Mantel tests were performed with XLSTAT 14 (Addinsoft) to determine Spearman correlations of proximity matrices calculated using the Bray-Curtis dissimilarity algorithm.

### Correlations with environmental variables.

The relationships between environmental data and the relative abundances of studied bacterial and eukaryotic groups were analyzed by Bray-Curtis dissimilarity distance-based linear models (DistML) (72) in the PERMANOVA+ add-on package of the PRIMER7 software (Primer Ltd., Plymouth, UK). Environmental variables were normalized, and a correlation matrix for the whole set was calculated. From the variables that were strongly correlated (the absolute value of the correlation coefficient was >0.7), only one was chosen for further analysis. Analyses were performed on untransformed relative abundance data using a stepwise selection procedure, and the best model was selected based on the statistical significance (9,999 permutations) and the values of the Akaike’s information criterion (AIC) and the Bayesian information criterion (BIC).

### Data availability.

The eukaryotic HTS data obtained with the general primers (TAReuk454FWD1 and TAReukREV3) were deposited in the ENA database under BioProject no. PRJEB23971, and those obtained with primers TAReuk454FWD1 and HaptoR1 were deposited under BioProject no. PRJEB31858. Bacterial HTS data from Jiřická Pond were deposited in NCBI as BioSamples SAMN11974970 to SAMN11974993 as part of BioProject PRJNA547706, and those from Lake Zurich were deposited under BioProject no. PRJNA545726.
