# Supplementary figures and images for: Bacterial and Eukaryotic Small-Subunit Amplicon Data Do Not Provide a Quantitative Picture of Microbial Communities, but They Are Reliable in the Context of Ecological Interpretations
Source: mSphere. 2020 Mar 4;5(2):e00052-20. doi: 10.1128/mSphere.00052-20 (PMC7056804; doi:10.1128/mSphere.00052-20)

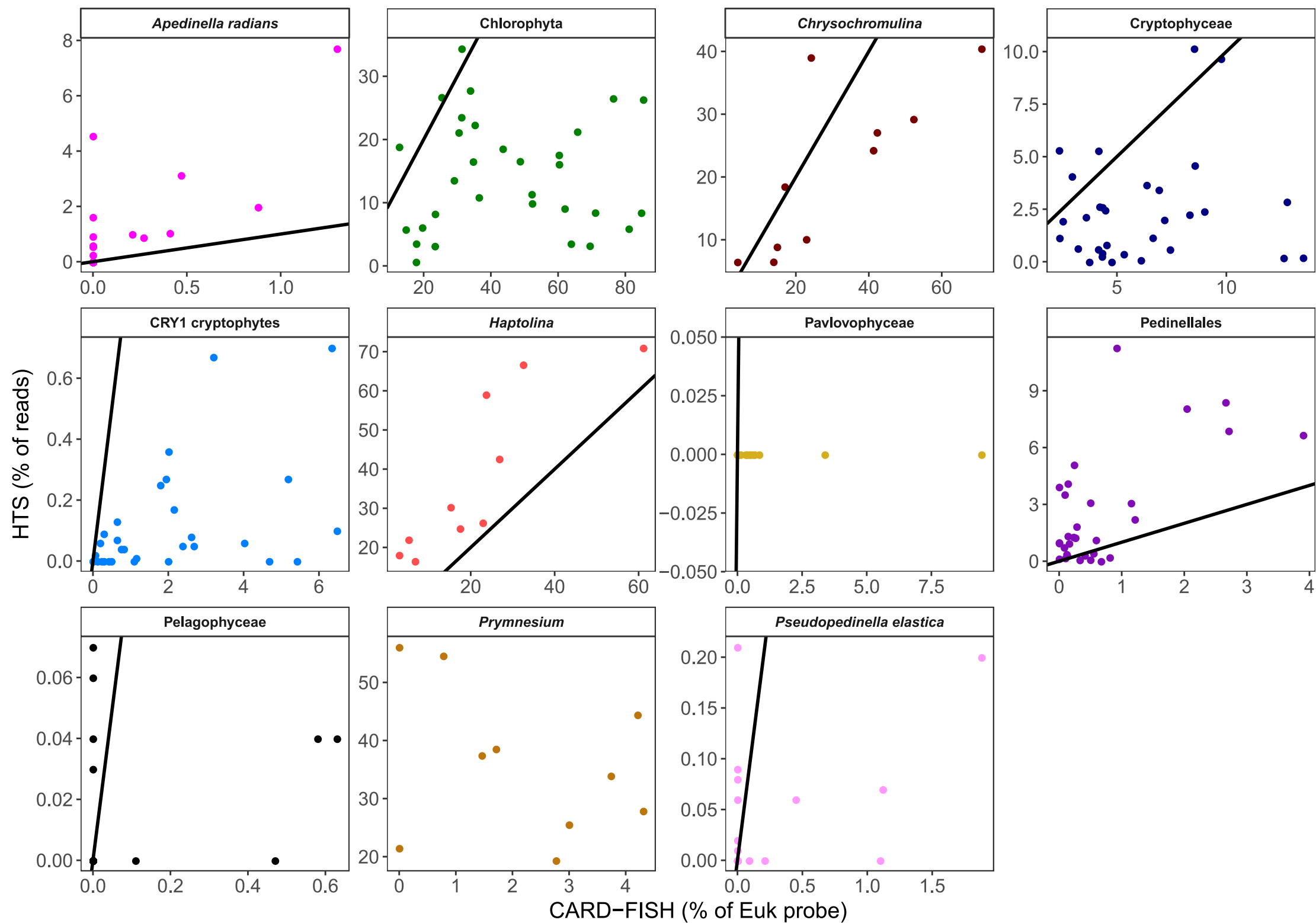

Supplement: FIG S1 [file mSphere.00052-20-sf001.pdf]

HTS (% of reads)

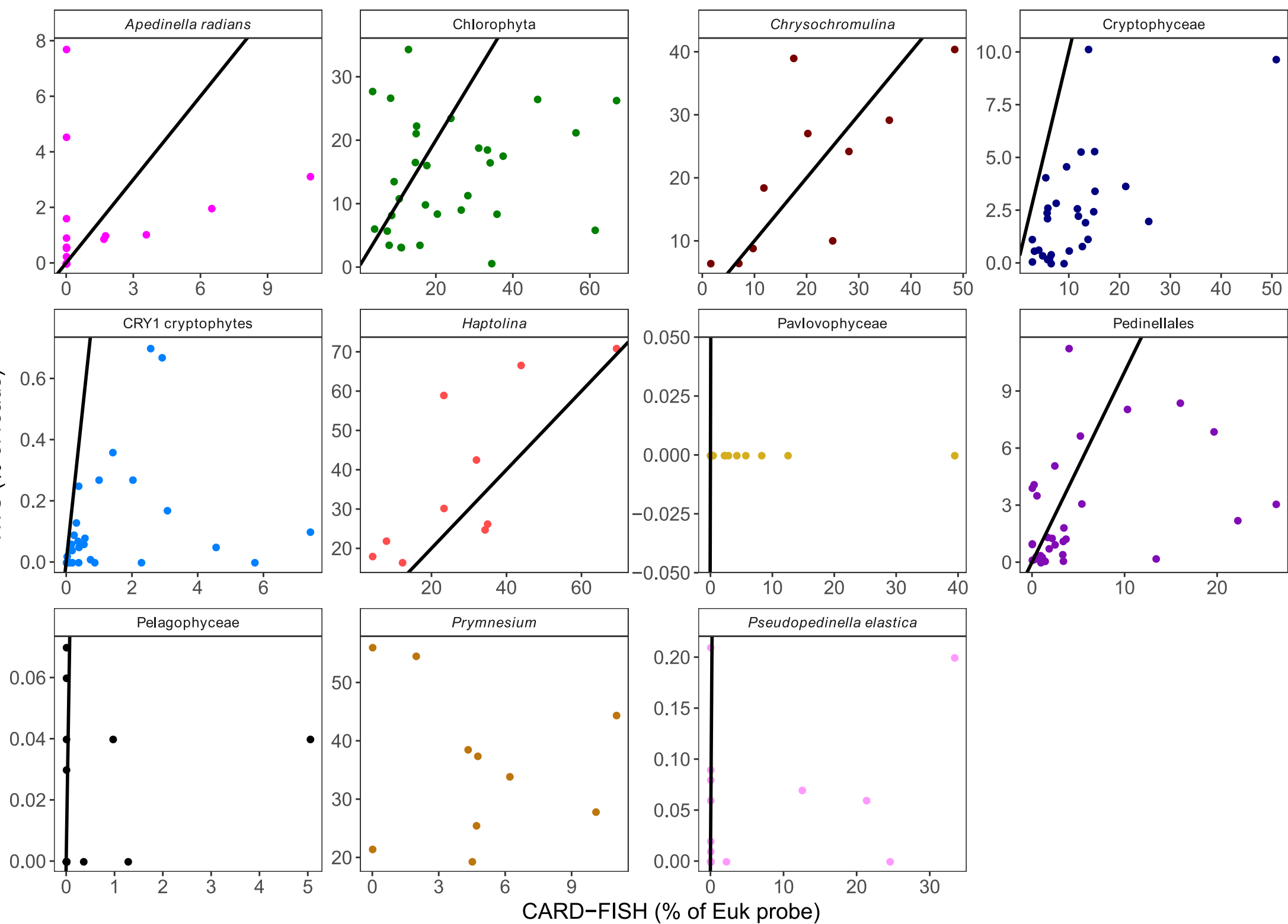

Supplement: FIG S3 [file mSphere.00052-20-sf003.pdf]

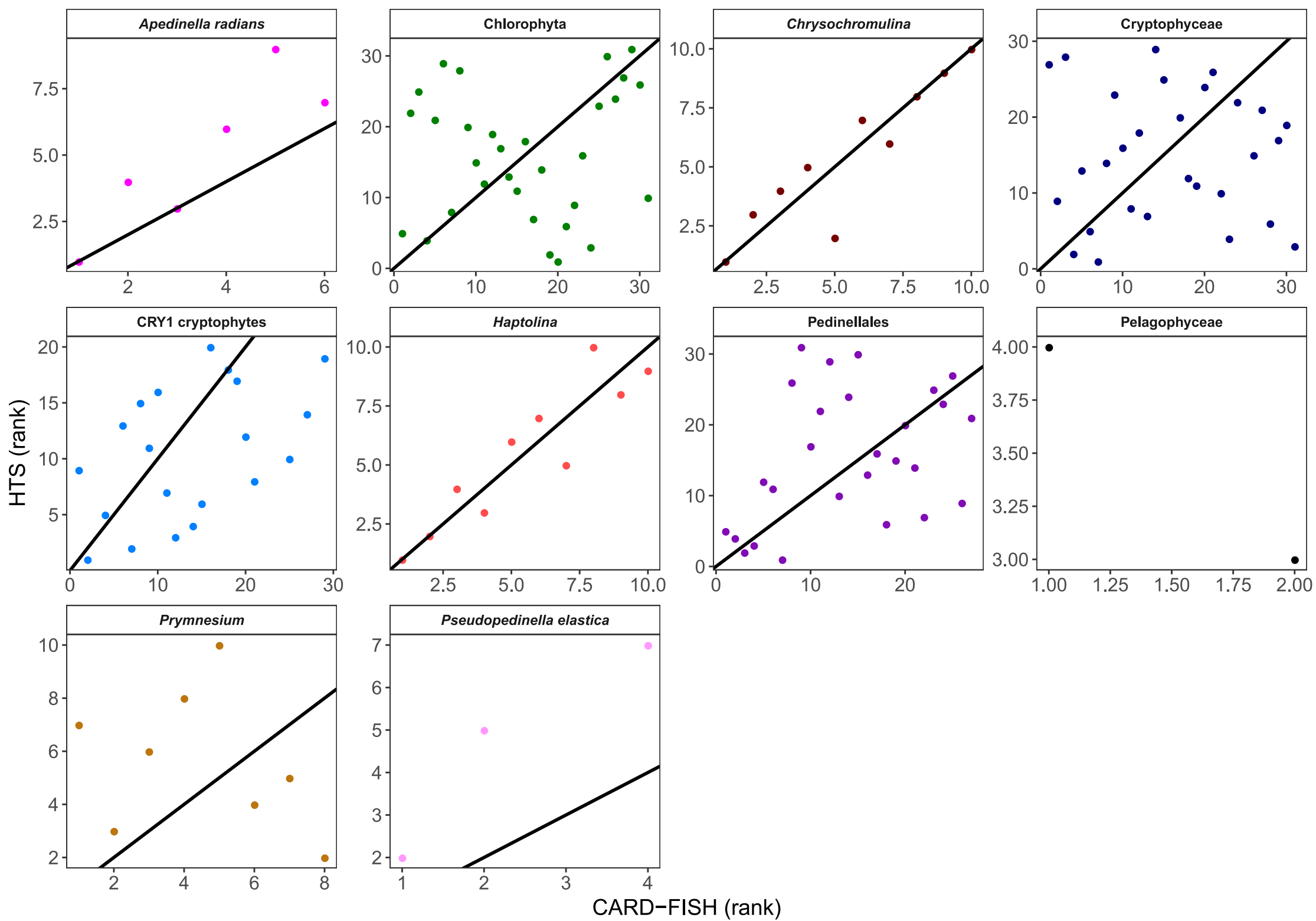

Supplement: FIG S4 [file mSphere.00052-20-sf004.pdf]

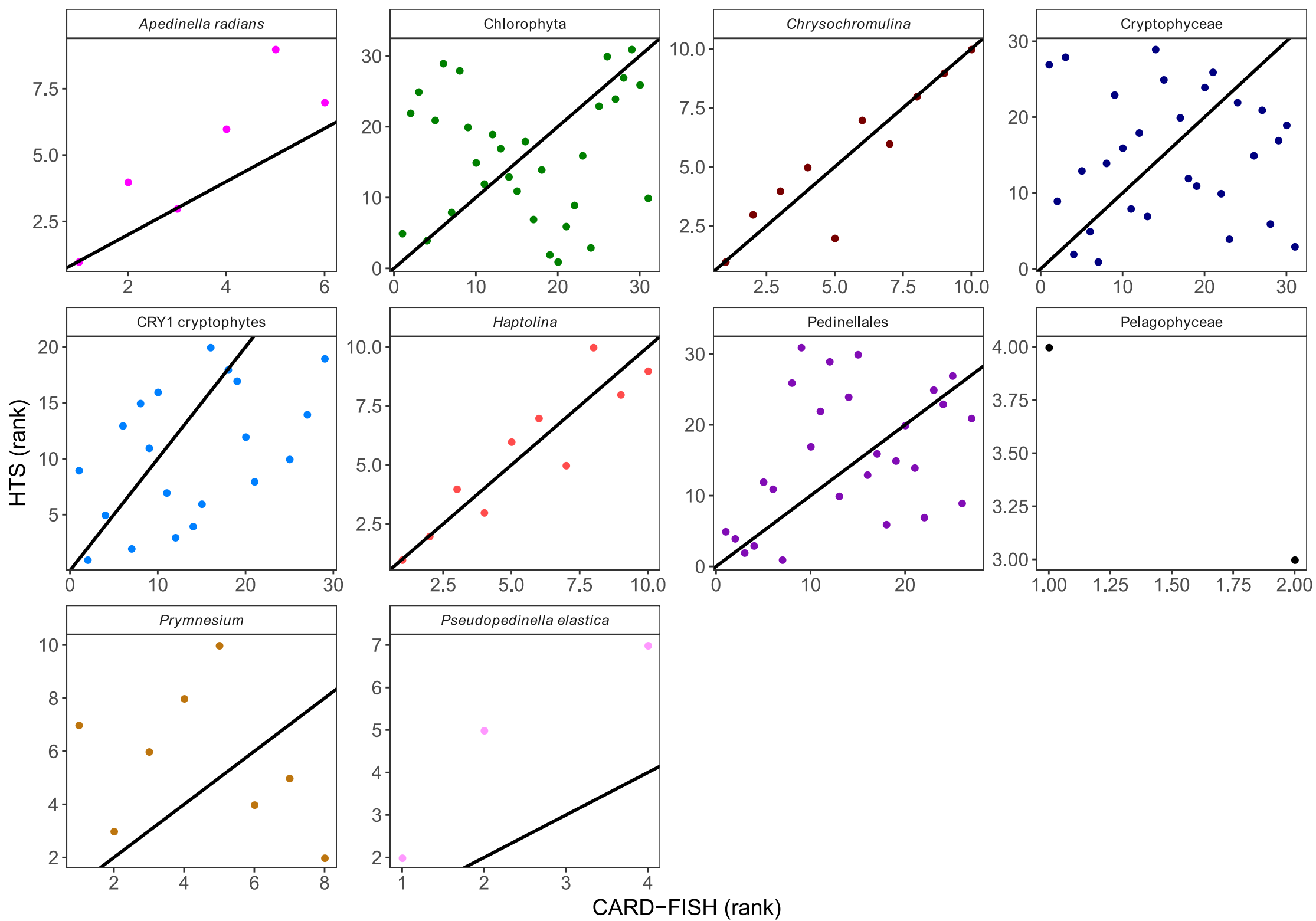

Supplement: FIG S5 [file mSphere.00052-20-sf005.pdf]

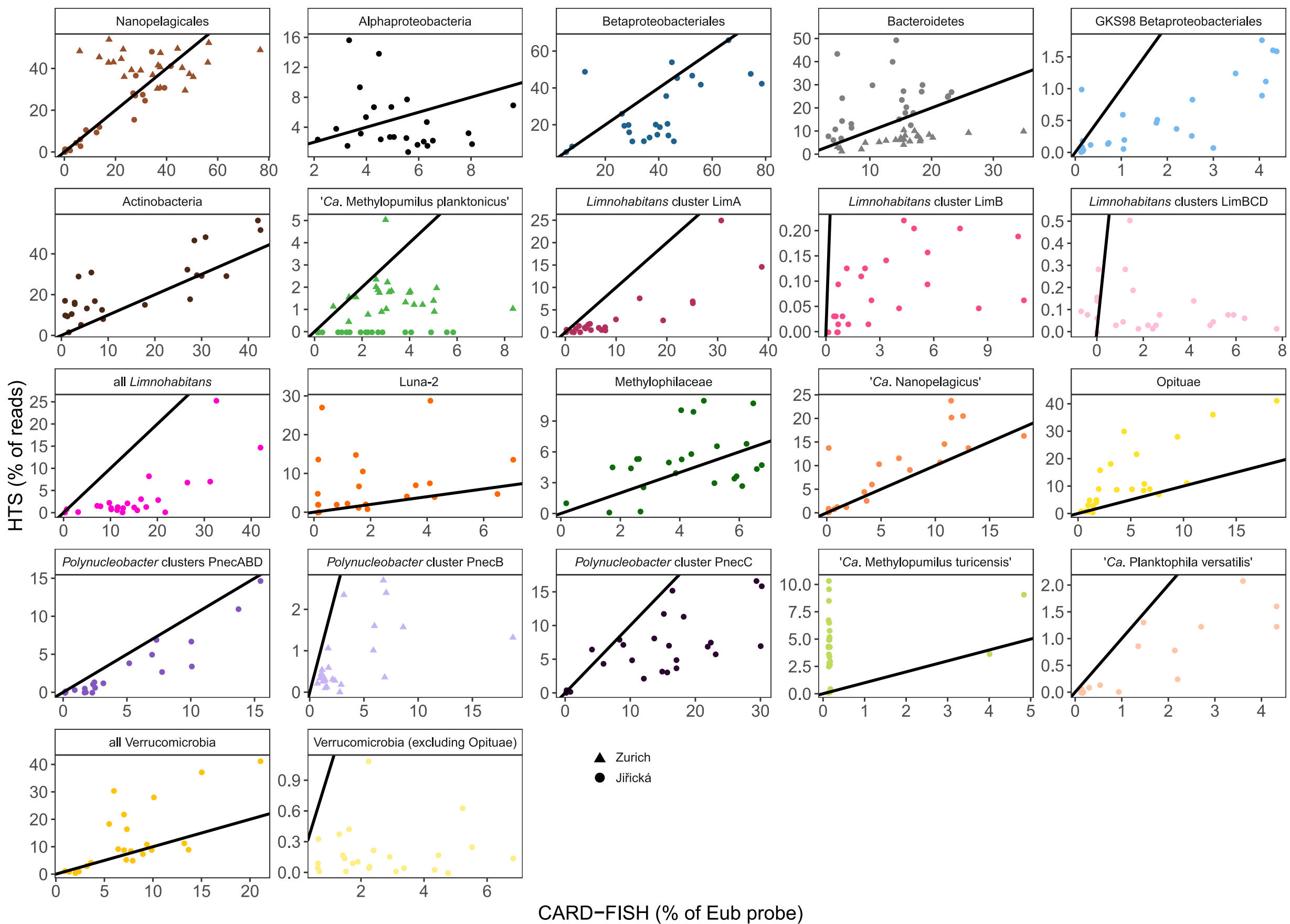

Supplement: FIG S6 [file mSphere.00052-20-sf006.pdf]

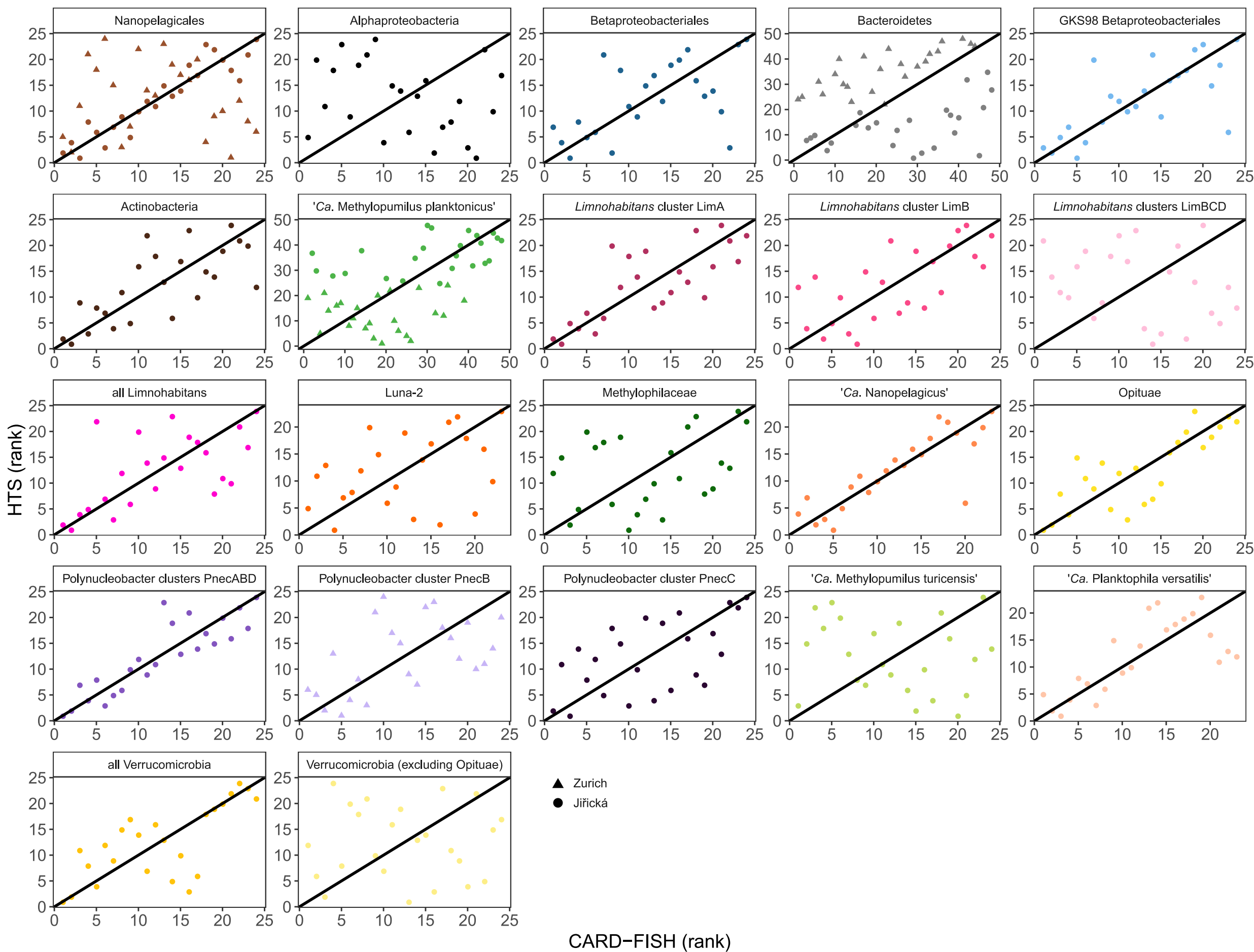

Supplement: FIG S7 [file mSphere.00052-20-sf007.pdf]

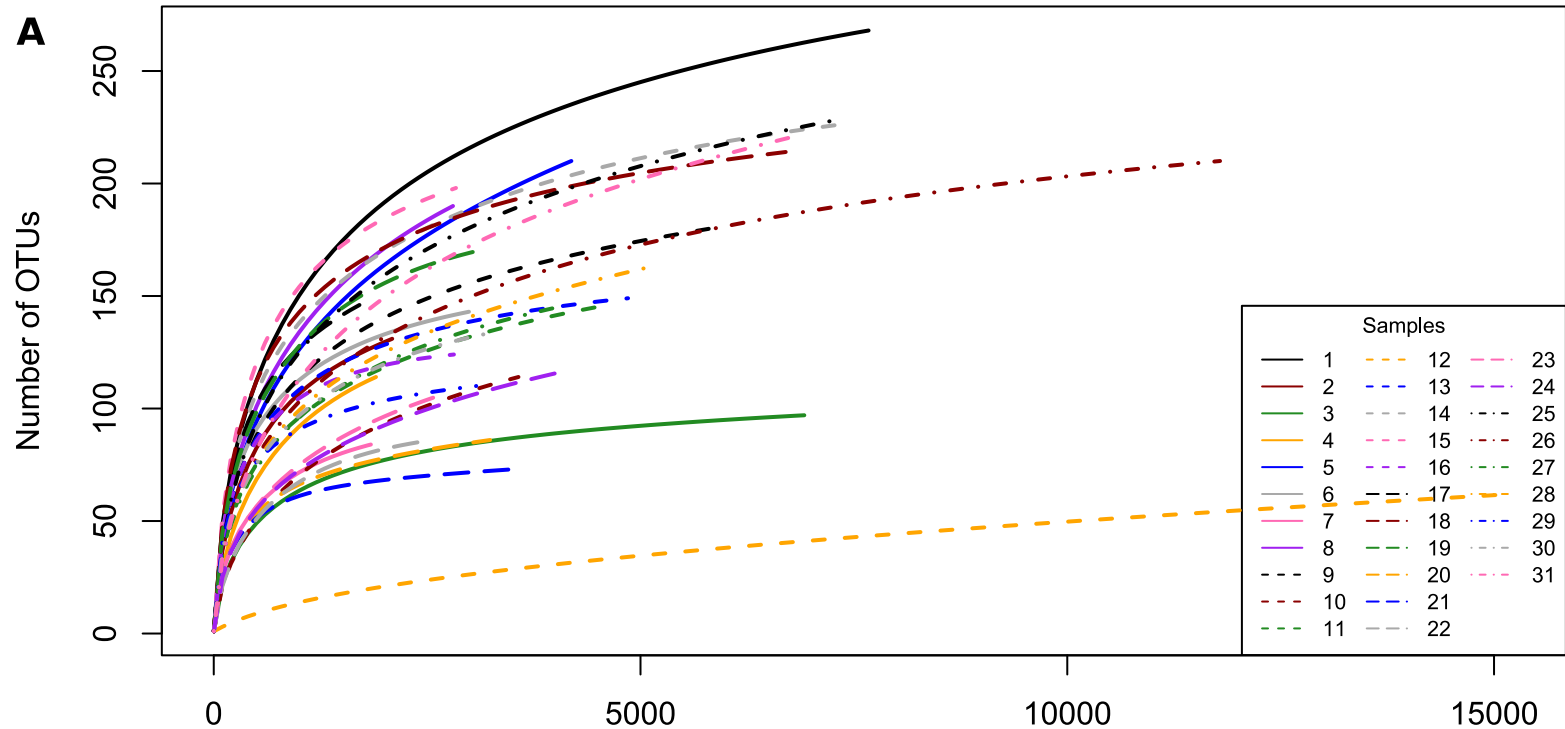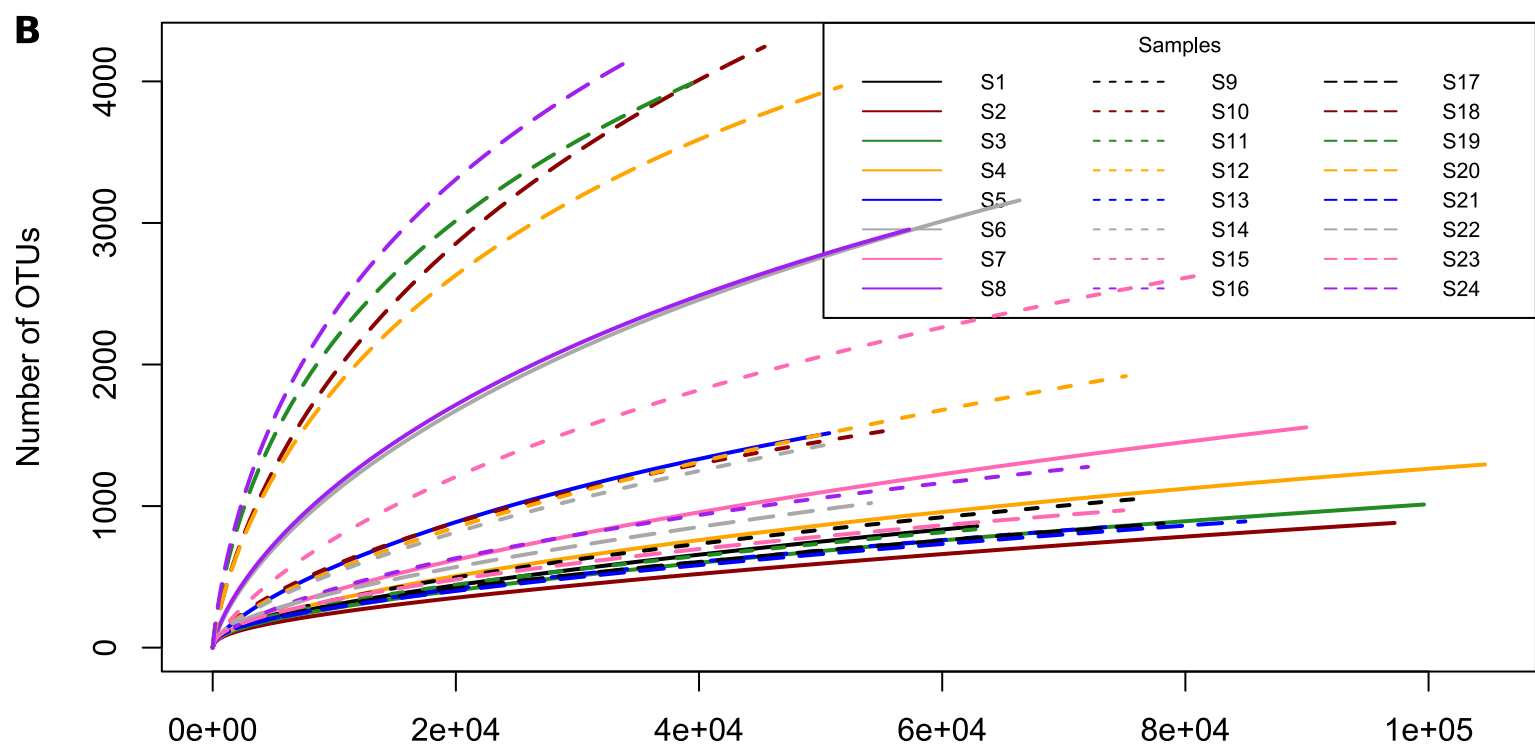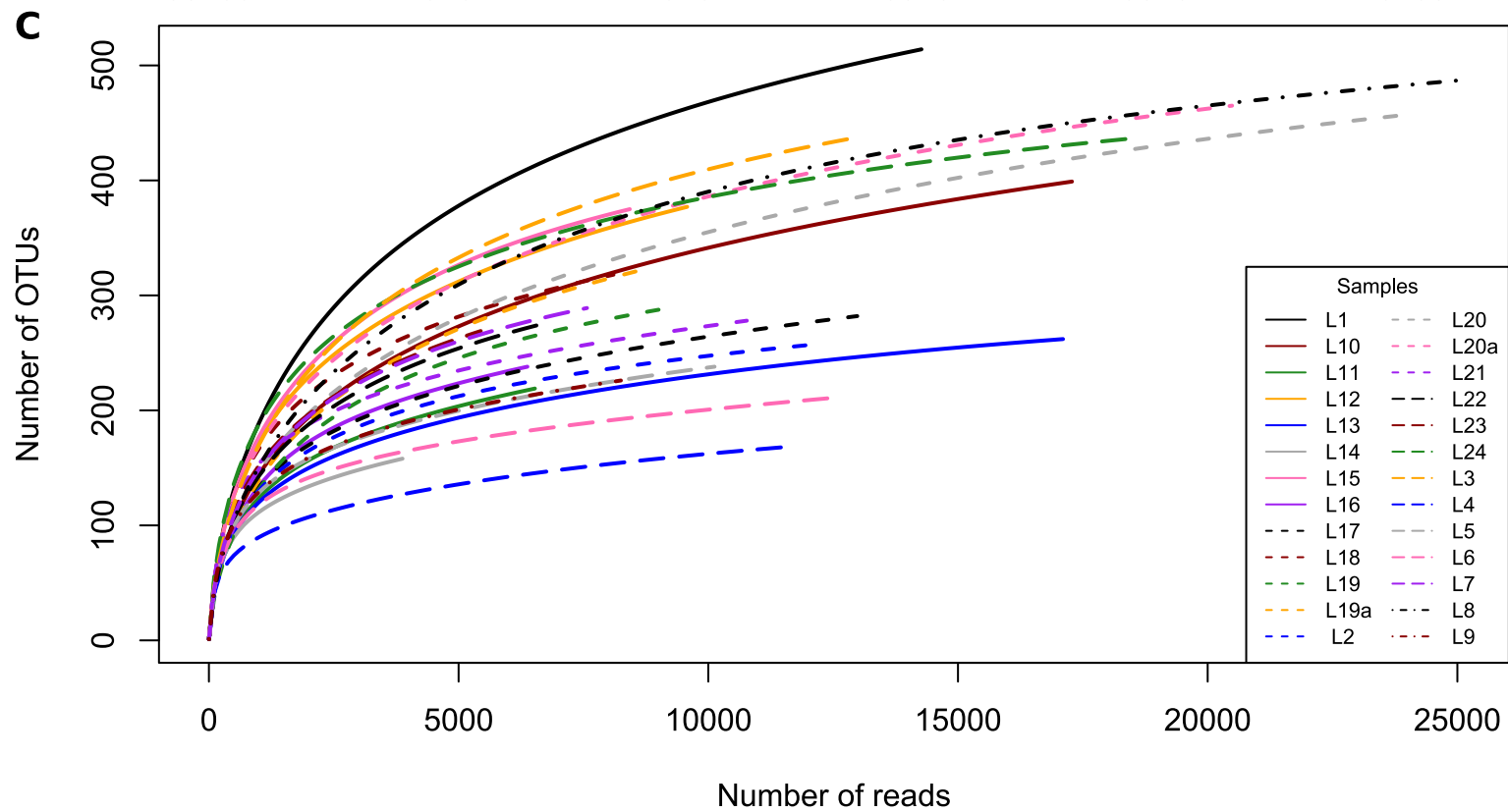

Supplement: FIG S8 [file mSphere.00052-20-sf008.pdf]
