# Supplementary material for: Bacterial and Eukaryotic Small-Subunit Amplicon Data Do Not Provide a Quantitative Picture of Microbial Communities, but They Are Reliable in the Context of Ecological Interpretations
Source: mSphere. 2020 Mar 4;5(2):e00052-20. doi: 10.1128/mSphere.00052-20 (PMC7056804; doi:10.1128/mSphere.00052-20)
Supplement: FIG S2 [file mSphere.00052-20-sf002.pdf]

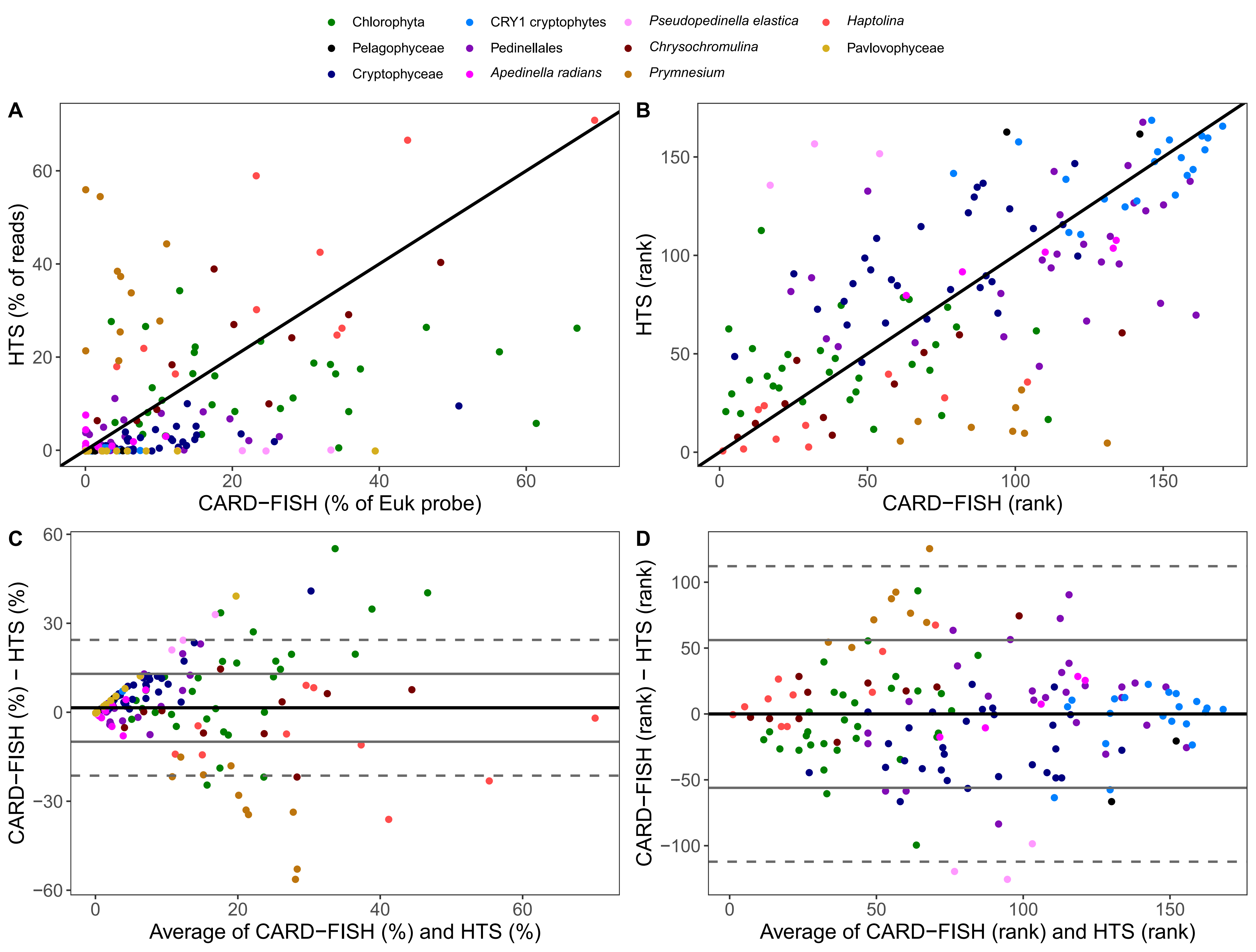
Chlorophyta
Pelagophyceae
Cryptophyceae
CRY1 cryptophytes
Pedinellales
Apedinella radians
Pseudopedinella elastica
Chrysochromulina
Prymnesium
Haptolina
Pavlovophyceae
A
HTS (% of reads)
CARD−FISH (% of Euk probe)
B
HTS (rank)
CARD−FISH (rank)
C
CARD−FISH (%) − HTS (%)
Average of CARD−FISH (%) and HTS (%)
D
CARD−FISH (rank) − HTS (rank)
Average of CARD−FISH (rank) and HTS (rank)
