# Supplementary material for: Bacterial and Eukaryotic Small-Subunit Amplicon Data Do Not Provide a Quantitative Picture of Microbial Communities, but They Are Reliable in the Context of Ecological Interpretations
Source: mSphere. 2020 Mar 4;5(2):e00052-20. doi: 10.1128/mSphere.00052-20 (PMC7056804; doi:10.1128/mSphere.00052-20)
Supplement: TABLE S1 [file mSphere.00052-20-st001.pdf]

Supplementary Table 1. List of primers used in this study.

Primer	Target group	Sequence (5'→ 3')	Reference
TAReuk454FWD1[FWD]	All eukaryotes	CCA GCA SCY GCG GTA ATT CC	Stoeck et al. (2010)
TAReukREV3[REV]	All eukaryotes	ACT TTC GTT CTT GAT YRA	Stoeck et al. (2010)
HaptoR1[REV]	Haptophyta	CGA AAC CAA CAA AAT AGC AC	Egge et al. (2013)
341F [FWD]	Bacteria	CCT ACG GGA GGC AGC AG	Muyzer et al. (1993)
907R [REV]	Bacteria	CCG TCA ATT CMT TTG AGT TT	Lane et al. (1985)
27F [FWD]	Bacteria	AGR GTT YGA TYM TGG CTC AG	Alm et. al (1996)
Uni522R [REV]	Bacteria	GWA TTA CCG CGG CKG CTG	Alm et. al (1996)

[FWD] – forward primer, [REV] – reverse primer

References

Alm, E. W., Oerther, D. B., Larsen, N., Stahl, D. A., Raskin, L. (1996). The oligonucleotide probe database. Appl Environ Microbiol 62: 3557-3559.

Egge, E., Bittner, L., Andersen, T., Audic, S., de Vargas, C., and Edvardsen, B. (2013) 454 Pyrosequencing to Describe Microbial Eukaryotic Community Composition, Diversity and

Lane, D.J., Pace, B., Olsen, G.J., Stahl, D.A., Sogin, M.L., and Pace, N.R. (1985) Rapid determination of 16S ribosomal RNA sequences for phylogenetic analyses. *Proceedings of the National Academy of Sciences of the United States of America* **82**: 6955-6959.

Muyzer, G., Dewaal, E.C., and Uitterlinden, A.G. (1993) Profiling of complex microbial populations by denaturating gradient gel-electrophoresis analysis of polymerase chain reaction-amplified genes-coding for 16S ribosomoal-rRNA. *Applied and Environmental Microbiology* **59**: 695-700.

Stoeck, T., Bass, D., Nebel, M., Christen, R., Jones, M.D.M., Breiner, H.-W., and Richards, T.A. (2010) Multiple marker parallel tag environmental DNA sequencing reveals a highly complex eukaryotic community in marine anoxic water. *Molecular Ecology* **19**: 21-31.
