# Supplementary material for: Bacterial and Eukaryotic Small-Subunit Amplicon Data Do Not Provide a Quantitative Picture of Microbial Communities, but They Are Reliable in the Context of Ecological Interpretations
Source: mSphere. 2020 Mar 4;5(2):e00052-20. doi: 10.1128/mSphere.00052-20 (PMC7056804; doi:10.1128/mSphere.00052-20)
Supplement: TABLE S2 [file mSphere.00052-20-st002.pdf]

Supplementary Table 2. List of probes, helpers and competitors used in this study. HB (%) – concentration of formamid in the hybridization buffer, T – hybridization temperature (washing temperature was 2°C higher), Probe: coverage (in percent of targeted sequences) of the probe, Primers 1: coverage of primers TAReuk454FWD1 and TAReukREV3 (Eukaryotes) or 341F and 907R (Bacteria, Lake Zurich), Primers 2: coverage of primers TAReuk454FWD1 and HaptoR1 (Eukaryotes) or 27F andUni522R (Bacteria, Jiřická Pond). The numbers are based on search in the reference Silva database release 132, conducted on 23 Nov 2019.

Probe	Target group	Sequence (5'→ 3')	HB (%)	T (^{o}C)	Probe	Primers 1	Primers2	Reference
				**EUKARYOTES**				
Chlo02	Chlorophyta	CTT CGA GCC CCC AAC TTT	40	35	85.4	79.2	0	Simon et al. (2000)
Pela01	Pelagophyceae	ACG TCC TTG TTC GAC GCT	40	35	91.8	95.9	0	Simon et al. (2000)
CryptB	Cryptophyceae	ACG GCC CCA ACT GTC CCT	50	46	60.9	90.0	0	Metfies and Medlin (2007)
CryptP_680	CRY1 cryptophytes	CAC AGT AAA CGA TCC GCG CAA	40	35	100	100	0	Piwosz et al. (2016)
Ped675	Pedinellales	TCA CAG TAA ACG ACA GGC GT	45	35	79.2	100	0	Piwosz and Pernthaler (2010)
Aperad631	*Apedinella radians*	CCA GCA TGA GTC CCC CTG AGG	45	46	100	100	0	Piwosz (2019)
Pseela1352	*Pseudopedinella elastica*	GCG AAG CAT TCC CAG CAC TAT	30	46	100	100	0	Piwosz (2019)
ChrysB2-Clade02	*Chrysochromulina*	AGT CGG GTC TTC CTG CAT GT	40	46	89.6	0	94.3	Simon et al. (1997)
PrymB1-Clade01	*Prymnesium*	GGA CTT CCG CCG ATC CCT AGT	50	46	70.3	0	87.2	Simon et al. (1997)
Haptol640	*Haptolina*	GGC AGA CCG GCA GGC AGG CCC	60	35	100	0	100	Piwosz (2019)
Pavlova01	Pavlovophyceae	CAC CTC TCT CTA CGG AAT	30	35	87.2	0	83.0	Eller et al. (2007)
				**BACTERIA**				
acI-853	Nanopelagicales (acI), Actinobacteria	AAT GCG TTA GCT GCG TCG CA	55	35	68.0	90.9	92.9	Warnecke et al. (2005)
acI-853-H	helpers for probe acI-853	**H1**: AAA CCG TGG AAG GTY CSC ACA ACT AG<br>**H2**: TCC CCA GGC GGG GCR CTT	55	35				Warnecke et al. (2005)
Alf968	Alphaproteobacteria	GGT AAG GTT CTG CGC GTT	55	35	75.9	87.4	83.7	Neef (1997)
BET42a	Betaproteobacteria	GCC TTC CCA CTT CGT TT	55	35	96.1	93.5	88.1	Manz et al. (1992)
BET42a-C	Competitor for probe BET42a	GCC TTC CCA CAT CGT TT	55	35				Manz et al. (1992)
CF968	Bacteroidetes	GGT AAG GTT CCT CGC GTA	55	35	94.2	87.0	86.7	Acinas et al. (2015)

Probe	Target group	Sequence (5'→ 3')	HB (%)	T (°C)	Probe	Primers 1	Primers2	Reference
GKS98-584	Uncult. lineage GKS98, Betaproteobacteria	ACC TCT CTT TCC GAA CCG CC	35	35	41.5	98.0	90.7	Salcher et al. (2013)
GKS98-584-C	Competitor for probe GKS98-584	AYC TYT CTT TCC GAA CCG CC	35	35				Salcher et al. (2013)
GKS98-584-H	Helpers for probe GKS98-584	**H1**: TGC GCA CGC TTT ACG CCC<br>**H2**: TAA GCC CTG GGA TTT CAC<br>**H3**: CGC TTT CGT GCA TGA GCG	35	35				Salcher et al. (2013)
HGC69a	Actinobacteria	TAT AGT TAC CAC CGC CGT	30	35	97.7	86.6	77.5	Roller et al. (1994)
LD28-1017	'*Ca*. Methylopumilus planktonicus', Betaproteobacteria	TCT CTT TCG AGC ACT TGA ACA	45	35	97.1	98.3	90.9	Salcher et al. (2011)
LD28-1017-C	Competitor for probe LD28-1017	TCT CTT TCG AGC ACT TTC ACA	45	35				Salcher et al. (2011)
LD28-1017-H	Helpers for probe LD28-1017	**H1**: TCT CTG CTC AAT TCG GTA<br>**H2**: CAG CAC CTG TGT TAC CGT<br>**H3**: GAG CTG ACG ACA GCC ATG<br>**H4**: CCC AAC ATC TCA CGA CAC	45	35				Salcher et al. (2011)
LimA-23S-1435	*Limnohabitans* cluster LimA, Betaproteobacteria	TCC AAC AGT CTG CTG AGC TAA CC	65	35	98.2	95.3	89.1	Shabarova et al. (2017)
LimA-23S-C	Competitor for probe LimA-23S-1435	TCC AAC AGT TGG CTG AGC TAA CC	65	35				Shabarova et al. (2017)
LimB-23S-920	*Limnohabitans* cluster LimB, Betaproteobacteria	CCC AAG CTG TAC TCG ACG GT	55	35	100.0	*	*	Shabarova et al. (in prep)
LimB-23S-C	Competitors for probe LimB-23S-920	**C1**: CCC AAG CTG TAC TCG TCG GT<br>**C2**: CCC AAG CTG TAC TCA ACG GT<br>**C3**: CCC AGG CTG TAC TCG ACG GT	55	35				Shabarova et al. (in prep)
R-BT065	*Limnohabitans* clusters LimBCD, Betaproteobacteria	GTT GCC CCC TCT ACC GTT	55	35	54.0	92.7	90.6	Šimek et al. (2001)
luna2-23S-1239	Luna2-cluster, Microbacteriaceae, Actinobacteria	CCT GAA TTC TCA CTC GTG TGG C	30	35	100.0	94.8	93.9	Shabarova et al. (in prep)

Probe	Target group	Sequence (5'→ 3')	HB (%)	T (°C)	Probe	Primers 1	Primers2	Reference
luna2-23S-C	Competitors for probe luna2-23S-1239	**C1**: CCT GAA TTC TCA CTC GTG TAG C<br>**C2**: CCT GCA TTC TCA CTC GTG TGG C	30	35				Shabarova et al. (in prep)
luna2-23S-H	Helpers for probe luna2-23S-1239	**H1**: TCA CYC BBM WTT CGC TAC TCA TK<br>**H2**: CGC CAC RKA TCR CTA ACT CYC AGG MTC ATT C<br>**H3**: RTC CAC GGC YGG DYM CHC CMC C	30	35				Shabarova et al. (in prep)
MET1217	Methylophilaceae, Betaproteobacteria	TTA CGT GTG AAG CCC TGG	40	35	95.0	92.5	83.3	Friedrich et al. (2003)
MET1217-C	Competitor for probe MET1217	TGA CGT GTG AAG CCC TGG	40	35				Friedrich et al. (2003)
MET1217-H	Helpers for probe MET1217	**H1**: CAA GGC CAG GTA AGG<br>**H2**: CCA TAA GGG CCA TGA GGA<br>**H3**: GTA CCG ACC ATW GTA	40	35				Friedrich et al. (2003)
Npel-23S-2669	'*Ca*. Nanopelagicus' (acI-B1), Nanopelagicales, Actinobacteria	ACA AGA GGT TCG TCC GTC C	60	35	100.0	93.3	93.8	Neuenschwander et al. (2018)
Npel-23S-C	Competitors for probe Npel-23S-2669	**C1**: ACY AGA GGT TCG TCC GTC C<br>**C2**: ACA AGA GGT TCG TCC ATC C<br>**C3**: ACY AGA GGT TCG TCC ATC C	60	35				Neuenschwander et al. (2018)
Npel-23S-H	Helpers for probe Npel-23S-2669	**H1**: CGG TCC TCT CGT ACT AGG GAC AGC<br>**H2**: GTG CTY CTG GCG RAA CAA CCG ACA C<br>**H3**: CYT TCC RAA CGT TGC WAA TCG GCC	60	35				Neuenschwander et al. (2018)
opitu-346	Opitutae, Verrucomicrobia	ATT CGA AAC TGC TGC CAC C	30	35	96.7	0.1	92.2	Shabarova et al. (in prep)
optiu-346-C	Competitor for opitu-346	ATT CCA AAC TGC TGC CAC C	30	35				Shabarova et al. (in prep)
PnecABD-445	*Polynucleobacter* clusters PnecABD, Betaproteobacteria	GAG CTG CTG TTT CTT CCC	55	35	78.5	85.6	90.9	Hahn et al. (2005)

Probe	Target group	Sequence (5'→ 3')	HB (%)	T (°C)	Probe	Primers 1	Primers2	Reference
PnecB-23s-166	*Polynucleobacter* cluster PnecB, Betaproteobacteria	GTT CGC TCC ACA CAC CCT	60	35	100.0	84.0	80.0	Wu and Hahn (2006)
PnecC-445	*Polynucleobacter* cluster PnecC, Betaproteobacteria	GAG CCG GTG TTT CTT CCC	55	35	80.9	98.2	80.0	Hahn et al. (2005)
PrD-732	'*Ca*. Methylopumilus turicensis', Betaproteobacteria	TCA GTA TTA GTC CAG GGG GCT G	50	35	100.0	96.9	100.0	Salcher et al. (2015)
PrD-732-C	Competitor for probe PrD-732	TCA GTA TTA GSC CAG GGG GCT G	50	35				Salcher et al. (2015)
Pver-23S-1420	'*Ca*. Planktophila vernalis' (acI-A7), Nanopelagicales, Actinobacteria	AAC TAC TAC CAC ACC GGT TCG	55	35	100.0	100.0	100.0	Neuenschwander et al. (2018)
Pver-23S-C	Competitors for probe Pver-23S-1420	**C1**: AAC TAC TAC CAC ACC GGT TCA<br>**C2**: AAC TAC TAC CAC ACC GGG TCG<br>**C3**: AAC TAC TAC AAC ACC GGT TCG<br>**C4**: AAC TAC TAC AAC ACC GGG TCA	55	35				Neuenschwander et al. (2018)
Pver-23S-H	Helpers for probe Pver-23S-1420	**H1**: CAT TAG TGG RTT CGT YAT GGG CGA ATT A<br>**H2**: AGC CAT CCA CCC ACG CRG CTT CTG TGT CAC ACC ATT GCT T	55	35				Neuenschwander et al. (2018)
Ver46	Verrucomicrobia (excluding Opitutae)	CGA CTT GCA TGT CTT ATC CA	40	35	69.1	0.1	85.6	modified after Buckley and Schmidt (2001)
Ver46-H	Helper for probe Ver46	CGC CGC CAG CGT TCR TT	40	35				modified after Buckley and Schmidt (2001)

References

Acinas, S.G., Ferrera, I., Sarmento, H., Diez-Vives, C., Forn, I., Ruiz-Gonzalez, C. et al. (2015) Validation of a new catalysed reporter deposition-fluorescence in situ hybridization probe for the accurate quantification of marine Bacteroidetes populations. *Environ Microbiol* **17**: 3557-3569.

Amann, R.I., Binder, B.J., Olson, R.J., Chisholm, S.W., Devereux, R., and Stahl, D.A. (1990) Combination of 16S ribosomal-RNA-targeted oligonucleotide probes with flow-cytometry for analyzing mixed microbial populations. *Applied and Environmental Microbiology* **56**: 1919-1925.

Buckley, D.H., and Schmidt, T.M. (2001) Environmental factors influencing the distribution of rRNA from Verrucomicrobia in soil. *FEMS Microbiology Ecology* **35**: 105-112.

Eller, G., Toebe, K., and Medlin, L.K. (2007) Hierarchical probes at various taxonomic levels in the Haptophyta and a new division level probe for the Heterokonta. *Journal of Plankton Research* **29**: 629-640.

Friedrich, U., Van Langenhove, H., Altendorf, K., and Lipski, A. (2003) Microbial community and physicochemical analysis of an industrial waste gas biofilter and design of 16S rRNA-targeting oligonucleotide probes. *Environmental Microbiology* **5**: 183-201.

Fuller, N.J., Tarran, G.A., Cummings, D.G., Woodward, E.M.S., Orcutt, K.M., Yallop, M. et al. (2006) Molecular analysis of photosynthetic picoeukaryote community structure along an Arabian Sea transect. *Limnology and Oceanography* **51**: 2502-2514.

Giovannoni, S.J. (1991) The polymerase chain reaction. In *Nucleic Acids Techniques in Bacterial Systematics*. Stackebrandt, E., and Goodfellow, M. (eds). New York, USA: John Wiley & Sons, pp. 177–203.

Hahn, M.W., Pöckl, M., and Wu, Q.L. (2005) Low Intraspecific Diversity in a *Polynucleobacter* Subcluster Population Numerically Dominating Bacterioplankton of a Freshwater Pond. *Applied and Environmental Microbiology* **71**: 4539-4547.

Manz, W., Amann, R., Ludwig, W., Wagner, M., and Schleifer, K.H. (1992) Phylogenetic Oligodeoxynucleotide Probes for the Major Subclasses of Proteobacteria - Problems and Solutions. *Systematic and Applied Microbiology* **15**: 593-600.

Metfies, K., and Medlin, L. (2007) Refining cryptophyte identification with DNA-microarrays. *Journal of Plankton Research* **12**: 1071-1075.

Neef, A. (1997) Anwendung der in situ Einzelzell-Identifizierung von Bakterien zur Populationsanalyse in komplexen mikrobiellen Biozönosen. In: Technische Universität München.

Neuenschwander, S.M., Ghai, R., Pernthaler, J., and Salcher, M.M. (2018) Microdiversification in genome-streamlined ubiquitous freshwater Actinobacteria. *The Isme Journal* **12**: 185-198.

Piwosz, K. (2019) Weekly dynamics of abundance and size structure of specific nanophytoplankton lineages in coastal waters (Baltic Sea). *Limnology and Oceanography* **64:** 2172-2186.

Piwosz, K., and Pernthaler, J. (2010) Seasonal population dynamics and trophic role of planktonic nanoflagellates in coastal surface waters of the Southern Baltic Sea. *Environmental Microbiology* **12**: 364-377.

Piwosz, K., Kownacka, J., Ameryk, A., Zalewski, M., and Pernthaler, J. (2016) Phenology of cryptomonads and the CRY1 lineage in a coastal brackish lagoon (Vistula Lagoon, Baltic Sea). *Journal of Phycology* **52**: 626–637

Roller, C., Wagner, M., Amann, R., Ludwig, W., and Schleifer, K.H. (1994) In situ probing of Gram-positive bacteria with high DNA G+C content using 23S rRNA-targeted oligonucleotides. *Microbiology* **140**: 2849-2858.

Salcher, M.M., Posch, T., and Pernthaler, J. (2013) In situ substrate preferences of abundant bacterioplankton populations in a prealpine freshwater lake. *Isme Journal* **7**: 896-907.

Salcher, M.M., Pernthaler, J., Frater, N., and Posch, T. (2011) Vertical and longitudinal distribution patterns of different bacterioplankton populations in a canyon-shaped, deep prealpine lake. *Limnology and Oceanography* **56**: 2027-2039.

Salcher, M.M., Neuenschwander, S.M., Posch, T., and Pernthaler, J. (2015) The ecology of pelagic freshwater methylotrophs assessed by a high-resolution monitoring and isolation campaign. *The Isme Journal* **9**: 2442.

Shabarova, T., Kasalický, V., Šimek, K., Nedoma, J., Znachor, P., Posch, T. et al. (2017) Distribution and ecological preferences of the freshwater lineage LimA (genus *Limnohabitans*) revealed by a new double hybridization approach. *Environmental Microbiology* **19**: 1296-1309.

Šimek, K., Pernthaler, J., Weinbauer, M.G., Horňák, K., Dolan, J.R., Nedoma, J. et al. (2001) Changes in bacterial community composition and dynamics and viral mortality rates associated with enhanced flagellate grazing in a mesoeutrophic reservoir. *Applied and Environment Microbiology* **67**: 2723-2733.

Simon, N., Brenner, J., Edvardsen, B., and Medlin, L.K. (1997) The identification of *Chrysochromulina* and *Prymnesium* species (Haptophyta, Prymnesiophyceae) using fluorescent or chemiluminescent oligonucleotide probes: a means for improving studies on toxic algae. *European Journal of Phycology* **32**: 393-401.

Simon, N., Campbell, L., Ornolfsdottir, E., Groben, R., Guillou, L., Lange, M., and Medlin, L.K. (2000) Oligonucleotide Probes for the Identification of Three Algal Groups by Dot Blot and Fluorescent Whole-Cell Hybridization. *Journal of Eukaryotic Microbiology* **47**: 76-84.

Warnecke, F., Sommaruga, R., Sekar, R., Hofer, J.S., and Pernthaler, J. (2005) Abundances, identity, and growth state of actinobacteria in mountain lakes of different UV transparency. *Applied and Environmental Microbiology* **71**: 5551-5559.

Wu, Q.L., and Hahn, M.W. (2006) Differences in structure and dynamics of *Polynucleobacter* communities in a temperate and a subtropical lake, revealed at three phylogenetic levels. *FEMS Microbiol Ecol* **57**: 67-79.
